# Identifying the E2F3-MEX3A-KLF4 signaling axis that sustains cancer cells in undifferentiated and proliferative state

**DOI:** 10.7150/thno.76619

**Published:** 2022-09-25

**Authors:** Xu Yang, Guilin Li, Yuhua Tian, Xin Wang, Jiuzhi Xu, Ruiqi Liu, Min Deng, Chunlei Shao, Yuwei Pan, Xi Wu, Mengzhen Li, Chaowei Zhang, Rui Liu, Jun Qin, Chen Zhang, Zhanju Liu, Xin Wu, Maksim V. Plikus, Christopher J. Lengner, Zongheng Zheng, Cong Lv, Zhengquan Yu

**Affiliations:** 1 State Key Laboratory of Agrobiotechnology, College of Biological Sciences, China Agricultural University, Beijing 100193, China.; 2 College of Agriculture and Life Sciences, Ankang University, Ankang, Shaanxi 725000, China.; 3 Department of Animal Biology, School of Veterinary Medicine, University of Pennsylvania, Philadelphia, Pennsylvania 19104, USA.; 4 Key Laboratory of Precision Nutrition and Food Quality, Ministry of Education, Department of Nutrition and Health, China Agricultural University, Beijing 100193, China.; 5 Department of Gastrointestinal Surgery, The Third Affiliated Hospital of Sun Yat-sen University, Guangzhou 510630, China.; 6 Clinical Laboratory, The Second Affiliated Hospital of Kunming Medical University, Kunming, Yunnan 650101, China.; 7 CAS Key Laboratory of Tissue Microenvironment and Tumor, CAS Center for Excellence in Molecular Cell Science, Shanghai Institute of Nutrition and Health, Chinese Academy of Sciences, Shanghai 200031, China.; 8 Department of Neurobiology, School of Basic Medical Sciences, Beijing Key Laboratory of Neural Regeneration and Repair, Capital Medical University, Beijing 100069, China.; 9 Department of Gastroenterology, The Shanghai Tenth People's Hospital, Tongji University, Shanghai 200072, China.; 10 State Key Laboratory of Reproductive Medicine, Nanjing Medical University, Nanjing, Jiangsu 211166, China.; 11 Department of Developmental and Cell Biology, Sue and Bill Gross Stem Cell Research Center, Center for Complex Biological Systems, University of California, Irvine, Irvine, CA 92697, USA.

**Keywords:** Colorectal cancer, Cancer stemness, MEX3A, KLF4, Radio-resistance

## Abstract

**Rationale:** Dysregulation of signaling that governs self-renewal and differentiation of intestinal stem cells (ISCs) is a major cause of colorectal cancer (CRC) initiation and progression.

**Methods:** qRT-PCR, western blotting, *in situ* hybridization, immunohistochemistry and immunofluorescence assays were used to detect the expression levels of MEX3A, KLF4 and E2F3 in CRC tissues. The biological functions of MEX3A were studied using *Mex3a* knockout (KO) and intestinal epithelium specific conditional knockout (cKO) mice, AOM-DSS mouse colorectal tumor model, Apc floxed mouse tumor model and intestinal and tumor organoids. Transcriptomic RNA sequencing (RNA-seq), RNA crosslinking immunoprecipitation (CLIP) and luciferase reporter assays were performed to explore the molecular mechanisms of MEX3A.

**Results:** RNA-binding protein MEX3A, a specific ISC marker gene, becomes ectopically upregulated upon CRC and its levels negatively correlate with patient survival prognosis. MEX3A functions as an oncoprotein that retains cancer cells in undifferentiated and proliferative status and it enhances their radioresistance to DNA damage. Mechanistically, a rate limiting factor of cellular proliferation E2F3 induces MEX3A, which in turn activates WNT pathway by directly suppressing expression of its pro-differentiation transcription factor KLF4. Knockdown of *MEX3A* with siRNA or addition of KLF4 agonist significantly suppressed tumor growth both by increasing differentiation status of cancer cells and by suppressing their proliferation.

**Conclusions:** It identifies E2F3-MEX3A-KLF4 axis as an essential coordinator of cancer stem cell self-renewal and differentiation, representing a potent new druggable target for cancer differentiation therapy.

## Introduction

Colorectal cancer (CRC) is the third most commonly diagnosed cancer type and the second leading cause of cancer-related deaths 1. Dysregulation of signaling that governs differentiation and self-renewal of intestinal stem cells (ISCs) is thought to be the driver of CRC initiation and progression 2. Aberrantly high activation of WNT pathway has been shown to drive self-renewal of cancer stem cells (CSCs), and WNT was shown to be overactivated in over 80% of CRC patients 3. Adenomatous polyposis coli (*APC*) gene is a WNT antagonist considered to be a gatekeeper of colorectal tumorigenesis. Mutations in *APC* commonly drive tumorigenic WNT over-activation in ISCs 4. In contrast, signals that govern ISC differentiation, such as BMP pathway, become downregulated in CRC 2, 5 and prolonged inactivation of BMP/SMAD4 signaling can result in intestinal adenoma development 6. However, it remains unresolved how upregulation in self-renewal signaling and downregulation in pro-differentiation signaling become coordinated upon CRC. Elucidating signaling molecules that control self-renewal and differentiation is not only fundamental for understanding the molecular mechanism of CRC, but it could also open new anti-cancer therapeutic opportunities. Indeed, the so-called cancer differentiation therapy, whereas highly malignant undifferentiated cancer cells become converted into differentiated cells of low tumorigenicity, has emerged as a promising strategy for cancer treatment with low side-effect profile 7.

CSCs are a stem cell-like subset of tumor cells that drive cancer initiation and its relapse following therapy 8, and they share many cellular and molecular features with normal ISCs. Both CSCs and ISCs a highly proliferative and capable of long-term clonogenicity 9. In terms of gene expression, ISCs exhibit WNT-related transcriptional signature, including *Lgr5* expression. LGR5 is also a widely-accepted CSC marker. Other shared stemness signature genes between ISCs and CSCs are *CD44*, *Smoc2* and *Ascl2* 10. To meet the need for rapid homeostatic turnover of the intestinal epithelium, most ISCs at the bottom of crypts are sustained in rapidly-dividing and undifferentiated state, whereas a subset of Lgr5^+^ ISCs move upward and enter the differentiation program 9. Analogous self-renewal and differentiation capacity has been revealed also in CSCs 10. Many signaling pathways governing self-renewal and differentiation, such as WNT, Notch, Hedgehog, BMP, MAPK and PI3K-AKT, shared by both ISCs and CSCs, albeit aberrant in CSCs 2. Increasing evidence indicates that many regulatory factors governing ISC self-renewal and differentiation are also critical for oncogenesis in colorectal cancer 11-13. Hence, elucidating gene regulatory network of ISC and CSC self-renewal and differentiation could inform of new therapeutic for CRC.

MEX3A is an evolutionarily conserved RNA-binding protein (RBP) belonging to the MEX-3 RBP family 14. MEX-3 maintains totipotency and modulates asymmetric blastomere division in *C. elegans* 15, 16. In *Xenopus* and in mice, Mex3a is required for neuroblast proliferation 17. Of particular relevance, Barriga *et al.* reported that *Mex3a* marks a reserve-like subset of Lgr5^+^ ISCs that are resistant to DNA damage 18. Another study demonstrated that Mex3a is critical for maintaining Lgr5^+^ ISC pool in developing intestine 19. Furthermore, recent reports have implicated *MEX3A* as an oncogene that promotes tumor growth and cancer cell invasion in a variety of cancers such as lung, breast and cervical cancer 20-22. In addition, *in vitro* assays showed that MEX3A promotes the migration, invasion and proliferation of CRC cells 23, 24. However, it is still unknown of the function and molecular mechanism of MEX3A in coordinating self-renewal and differentiation signals in CRC. In this study, we found that *MEX3A* becomes ectopically upregulated in CRC and correlates with low grade cancer differentiation and poor survival prognosis. Mechanistically, E2F3-induced MEX3A overexpression sustains cancer cells in a rapidly dividing and undifferentiated state by directly suppressing KLF4, a key pro-differentiation regulator, to activate WNT signaling. Thus, E2F3-MEX3A-KLF4 is an essential signaling axis coordinating CSC self-renewal and differentiation. Targeting MEX3A or KLF4 not only suppressed CRC cell proliferation, but also increased their differentiation status, suggesting that it might represent a new potent target for cancer differentiation therapy upon CRC.

## Materials and Methods

Additional information is provided in Supplementary Materials and Methods.

### Ethics

All animal procedures were authorized by the Beijing Laboratory Animal Management Committee. All animal studies were performed in strict adherence to the Institutional Animal Care and Use Committee (IACUC) guidelines of China Agricultural University (approval number: SKLAB-2015-04-03).

### Mice

*Villin-Cre* mice (stock number: T000142) were purchased from the National Resource Center of Model Mice. *Lgr5-EGFP-IRES-Cre^ERT2^* mice (stock number: 008875) and *Kras^LSL-G12D^* mice (stock number: 008179) were obtained from Jackson Laboratories. *Mex3a* KO mice were obtained from Xin Wu's laboratory at Nanjing Medical University. *Mex3a^flox/flox^* mice were generated at the Shanghai Model Organisms Center, and first exon (1328 bp) of *Mex3a* was targeted with flanking LoxP sites, resulting in two LoxP loci.

### Patients and clinical specimens

Human CRC tissue microarray was obtained from Jun Qin's laboratory at the Shanghai Institute of Nutrition and Health, Chinese Academy of Sciences. Microarray contains 78 pairs of benign colorectal tissues and cancer tissues and 13 cancer tissue samples. Detailed information on the microarray is summarized in **Table S1**. All patients were from the Department of Colorectal Surgery, Fudan University Shanghai Cancer Center (FUSCC). Use of clinical samples, as well as review of all pertinent patient records, were approved by the Ethical Committee and Institutional Review Board of FUSCC in compliance with ethical standards and patient confidentiality, and informed consent was obtained from all patients.

### Irradiation injury

Two-month-old adult mice were subjected to 12 Gy γ-radiation and sacrificed at the indicated timepoints to obtain ileum samples. Cultured HCT116 cells and mouse tumor organoids were subjected to 2 Gy and 8 Gy γ-radiation respectively and used for downstream experiments.

### AOM and DSS treatment

Two-month-old control and *Mex3a* cKO mice were intraperitoneally injected with AOM (Sigma-Aldrich) at a concentration of 10 mg/kg body weight. Five days after AOM injection, mice were treated with the so-called DSS cycle comprising two steps: the mice were fed 2% (w/v) DSS (molecular weight: 36 000-50 000, MP Biomedicals) for 5 days and then normal water for 14 days. Mice were subjected to a total of 3 DSS cycles. After treatment, mice were sacrificed to obtain distal colon tissues.

### CLIP-qPCR

HCT116 cells transfected with HA-tagged MEX3A expression plasmids were subjected to ultraviolet crosslink twice at 400 mJ/cm^2^ in a Stratalinker (Model 2400) and then lysed with 1 × PXL buffer (1 × PBS, 0.1% SDS, 0.5% deoxycholate, 0.5% NP-40) plus protease inhibitor and RNAsin. After centrifugation, supernatant was incubated with protein A Dynabeads (Thermo, 10002D), conjugated to anti-HA antibody (Santa, sc-7392) or normal mouse IgG (Santa, sc-2025) for 4 hours at 4°C. Beads were washed with 1 × PXL buffer/5 × PXL buffer (5 × PBS, 0.1% SDS, 0.5% deoxycholate, 0.5% NP-40)/PNK buffer (50 mM Tris-HCl pH 7.4, 10 mM Mgcl_2_ and 0.5% NP-40), and digested with Proteinase K. RNA was extracted from beads and then reverse transcripted using the SuperScript^TM^ IV Reverse Transcriptase synthesis system (Thermo, 18091050).

### Xenograft tumor

Five-week-old male nude mice (BALB/c nude) were obtained from Beijing Vital River Laboratory Animal Technology Co., Ltd. and maintained in specific pathogen-free conditions. Mice were randomly grouped and subcutaneously injected with 5.0×10^6^ treated HCT116 cells in a total volume of 100 μl PBS. Mice were sacrificed 3 weeks after transplantation, and tumor length, width and weight were measured. All tumors were fixed in 10% PFA and embedded in paraffin. Tumor sizes were calculated as 0.5 × length × width^2^.

### Statistical analysis

At least three biologically independent experiments were performed unless stated otherwise. All data are presented as the mean ± standard deviation (SD). The *P* value was obtained by unpaired two-tailed Student's t-test, and asterisks denote statistical significance (*P < 0.05; **P < 0.01; ***P < 0.001).

## Results

### *MEX3A* becomes ectopically upregulated in CRC and correlates with stemness

**Figure 1:**
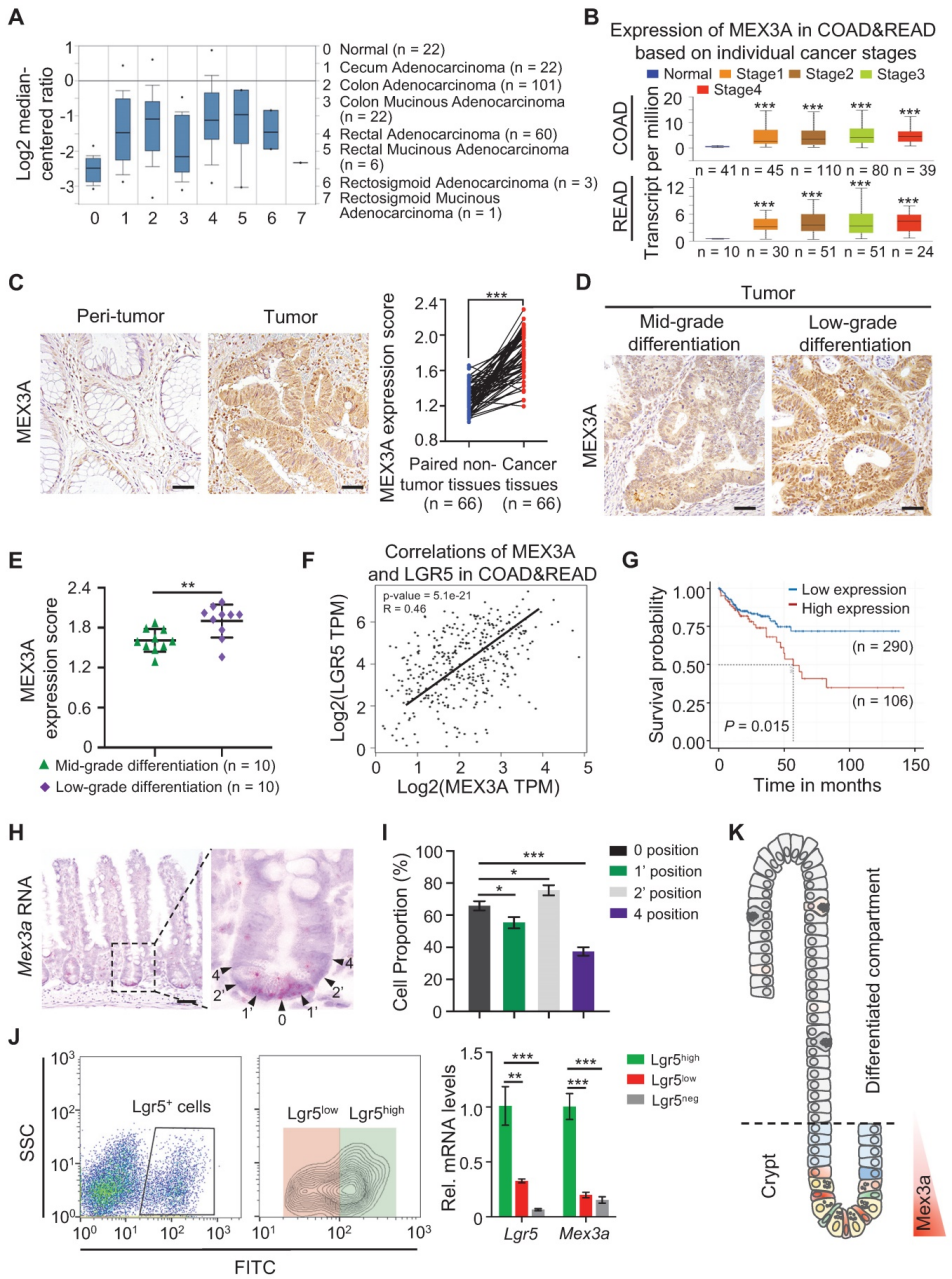
** MEX3A is ectopically overexpressed in CRC. A,** Analysis of TCGA database showing upregulation of *MEX3A* in different colorectal tumor types. n, number of patient samples per colorectal tumor type. **B,** *MEX3A* levels increase with cancer stages in CRC patients. n, number of patient samples per colorectal cancer stage. **C,** Representative immunohistochemical images of MEX3A in CRC tissue and its paired adjacent normal tissues based on the colorectal cancer tissue array. Scores of MEX3A expression levels were quantified. Scale bar: 50 μm. **D,** Immunohistochemistry for MEX3A in human CRC samples containing low-grade and mid-grade differentiation tumors. Scale bar: 50 μm. **E,** Scores of MEX3A expression levels were quantified in panel D. **F,** Spearman correlation analysis of MEX3A and LGR5 (*P* < 0.001; *R* = 0.46) in human CRC based on TCGA-COAD&READ database. **G,** Kaplan-Meier survival curve of 396 CRC patients. *P* = 0.015. **H,** *In situ* hybridization for *Mex3a* in intestinal crypts from 8-week-old mice. High-magnification of the outlined area is on the right. Arrowheads point to crypt cells at 0, 1', 2' and 4 position. Scale bar: 50 μm. **I,** Frequency of* Mex3a*^+^ cells at indicated positions of intestinal crypts is quantified based on 111 crypts from 3 mice. **J,** Representative flow cytometry images of Lgr5^neg^, Lgr5^low^ and Lgr5^high^ cells from intestinal crypts of *Lgr5^EGFP-CreERT2^* mice and qRT-PCR analysis for *Mex3a* and *Lgr5* in sorted Lgr5^neg^, Lgr5^low^ and Lgr5^high^ cells. n = 3 biological replicates. **K,** Schematic of *Mex3a* expression in the intestinal crypt-villus axis. Data are presented as the mean ± SD. *P < 0.05; **P < 0.01; ***P < 0.001.

To begin understanding the role of *MEX3A* in CRC, we analyzed its levels in different types of human CRC tissues from The Cancer Genome Atlas (TCGA) database. *MEX3A* levels were markedly elevated in almost all types of colorectal tumor tissues except rectosigmoid mucinous adenocarcinoma (**Figure 1A**), and they positively correlated with individual cancer stages (**Figure 1B**). Similarly, analysis of 66 colon adenocarcinoma/adjacent normal tissue pairs from individual patients from our cohort revealed consistent overexpression of MEX3A in colon tumors (**Figure 1C**). Significantly, MEX3A levels were higher in low differentiation grade tumors than in mid-grade counterparts (**Figure 1D-E**). Its levels in CRC tissues also consistently positively correlated with the levels of *LGR5,* a well-established marker for CSC (**Figure 1F**). This suggests potentially important role for MEX3A in cancer stem cells. Indeed, *MEX3A* was also upregulated in human colorectal cell lines compared to normal colon epithelial cells, and it was particularly high in HCT116 cells, which are mainly composed of stem-like cancer cells 25 (**Figure S1A-B**). Consistently, *MEX3A* levels correlated with poor survival probability in CRC patients from TCGA database (**Figure 1G**). In addition, *Mex3a* was also upregulated in mouse colitis-associated tumors from the azoxymethane-dextran sulfate sodium (AOM-DSS) model (**Figure S1C-E**). Collectivelly, these data support that *MEX3A* is ectopically upregulated in CRC and its level is positively correlated with low differentiation and high stemness state.

These correlations in CRC prompted us to examine *Mex3a* in normal ISCs under physiological conditions. We show that *Mex3a* is primarily expressed in crypt cells at positions 0, 1' and 2' and becomes notably reduced in cells residing at the +4 position and above (**Figure 1H-I**). Consistently, qRT-PCR and Western blotting show that *Mex3a* is upregulated in Lgr5^high^ ISCs *vs.* Lgr5^low^ or Lgr5^neg^ cells (**Figure 1J, S1F**). This expression pattern (**Figure 1K**) suggests a potential function for *Mex3a* in ISCs. Upon injury, *Mex3a* levels sharply increase in regenerative foci of intestinal crypts from 24 to 120 hours after 12 Gy of γ-radiation, when surviving ISCs are highly proliferative (**Figure S1G-I**). This suggests that *Mex3a* might be important for ISC-driven regeneration.

### E2F3 directly upregulates *MEX3A* upon CRC

**Figure 2:**
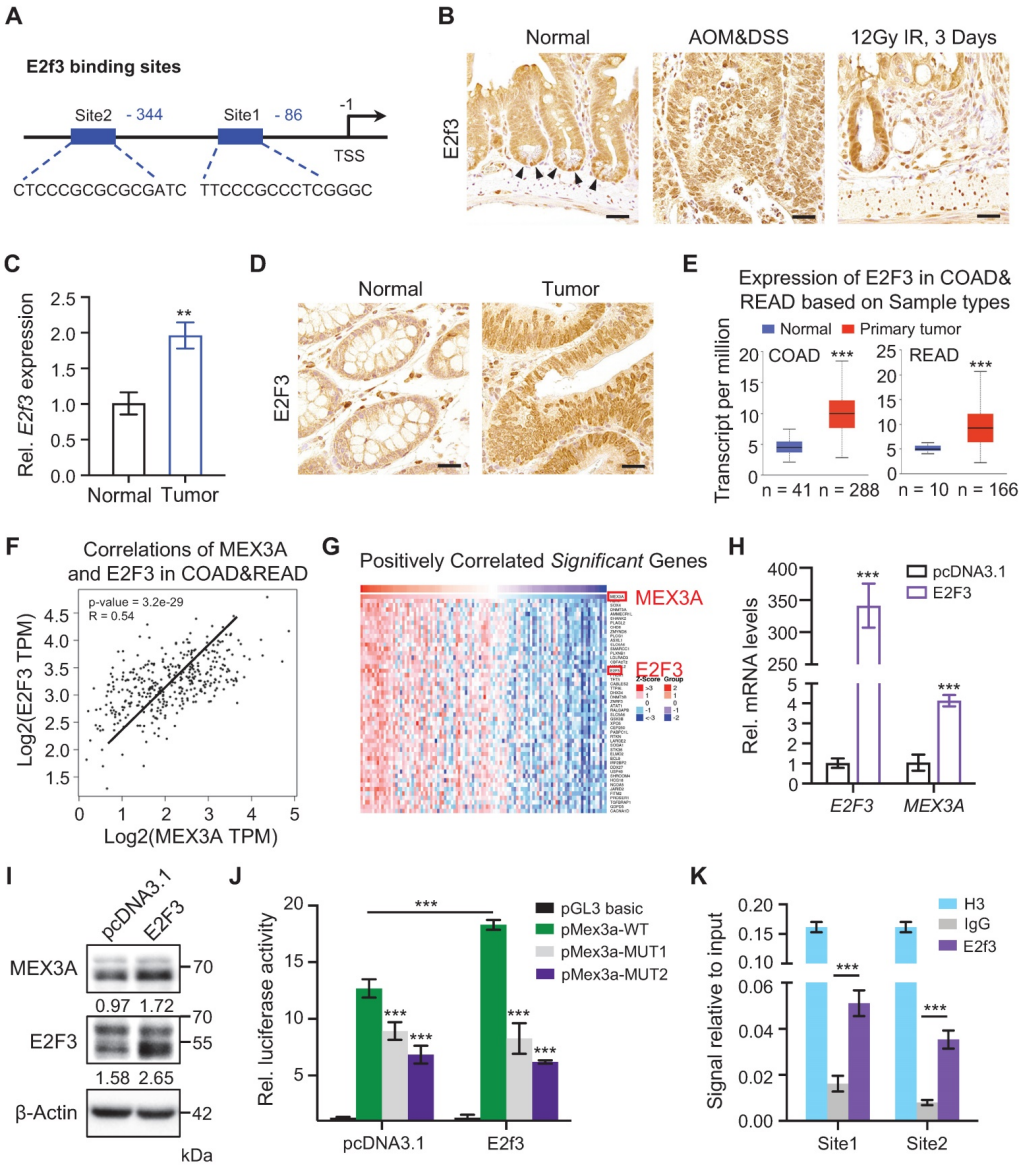
** E2f3 directly regulates *Mex3a* expression. A,** Schematic diagram showing two potential E2f3 binding sites at *Mex3a* promoter. **B,** Immunohistochemical staining for E2f3 in normal mouse intestinal crypts, AOM-DSS mouse colon tumors and regenerative foci in mouse intestinal crypts 3 days after 12 Gy γ-radiation. Arrowheads point to E2f3^+^ cells at the base of intestinal crypts. Scale bar: 25 μm. **C,** qRT-PCR analysis of *E2f3* in normal mouse colon tissues and AOM-DSS-induced mouse colon tumors. n = 3. **D,** Representative immunohistochemical images of E2F3 in human colorectal peritumor and tumor tissues from CRC patients. Scale bar: 25 μm. **E,** Box plots of *E2F3* expression in normal colorectum tissues and colorectal tumor tissues based on TCGA data. n, number of patient samples. **F,** Spearman correlation analysis of MEX3A and E2F3 (*P* < 0.001; *R* = 0.54) in human CRC based on TCGA database. **G,** Heatmap of genes significantly positively correlated with *MEX3A* in human CRC tissues based on TCGA database. The parameters of color key indicate the Z-score. **H,** qRT-PCR for *E2F3* and *MEX3A* in HCT116 cells treated with pcDNA3.1 empty vector or pcDNA3.1-E2F3 plasmids. n = 3. **I,** Western blotting for E2F3 and MEX3A in HCT116 cells treated with pcDNA3.1 empty vector or pcDNA3.1-E2F3 plasmids. β-Actin was used as loading control. **J,** Luciferase activity in lysates of CT26 cells transfected with luciferase reporter plasmids containing pGL3-basic empty vector, wild-type *Mex3a* promoter, or *Mex3a* promoter with mutations in E2f3 binding sites under normal and *E2f3* overexpression conditions. n = 3. **K,** Chromatin immunoprecipitation assay was carried out on CT26 cells using antibodies against E2f3 and Histone H3. The antibody against Histone H3 was used as a positive control. IgG was used as a negative control. The enrichment of E2f3 binding to *Mex3a* promoter was quantified using qRT-PCR. n = 3 technical replicates. Data are presented as the mean ± SD. *P < 0.05; **P < 0.01; ***P < 0.001.

To uncover upstream *Mex3a* regulators we used JASPAR database and analyzed putative transcription factor binding sites within 2 kb region upstream of *Mex3a* transcription start site. This yielded two binding sites for E2F3 (**Figure 2A**), which is a known oncogene with rate-limiting function in cell proliferation 26. Indeed, high E2F3 expression associates with poor survival and prognosis in various cancer types 27. Analogous to *Mex3a*, *E2f3* is also dramatically upregulated in AOM-DSS-induced mouse colon tumors, and is highly expressed in ISCs and in regenerative foci following irradiation (**Figure 2B-C**). E2F3 is also upregulated in human colorectal tumors (**Figure 2D**). Analysis of TCGA data also revealed a consistent increase in *E2F3* in human CRCs (**Figure 2E**). Moreover, positive association between E2F3 and MEX3A levels was found in CRC tissues (**Figure 2F-G**). Thus, the expression pattern of *E2f3*/*E2F3* in CRC and intestinal epithelium is highly similar to that of *Mex3a*/*MEX3A*.

To test whether E2F3 directly regulates *MEX3A*, we overexpressed *E2F3* in HCT116 cells and found that E2F3 induction significantly upregulates *MEX3A* both at the RNA and protein levels (**Figure 2H-I**). Furthermore, luciferase reporter assay revealed that E2F3 overexpression induces *MEX3A* promoter activity, whereas mutations in E2F3 binding sites blocked this activity (**Figure 2J**). Chromatin immunoprecipitation (ChIP) assays showed that E2f3 is recruited to its binding sites on the *Mex3a* promoter (**Figure 2K**). Collectively, these results suggest that E2F3 directly regulates *MEX3A* expression.

### Mex3a is required for ISC stemness and proliferation

In order to understand *in vivo* functions of Mex3a under physiological conditions and during tumorigenesis, we first generated constitutive *Mex3a* knock-out (KO) mice (**Figure S2A**), in which exon 1 and partial exon 2 were deleted. *Mex3a* was efficiently deleted in intestines of KO mice (**Figure S2B-C**). Body weight of KO mice was generally lower than in littermate controls (**Figure S2D**). Deletion of *Mex3a* led to a significant reduction in crypt depth with fewer proliferative cells, yet it had no noticeable effect on villus length (**Figure S2E-F**). Alkaline phosphatase has been used extensively as a marker for enterocyte differentiation 28. We found that alkaline phosphatase signal was stronger in villi from *Mex3a* KO mice than in villi from littermate controls (**Figure S2G**), suggesting excessive differentiation. Deletion of *Mex3a* also led to a significant increase in the number of goblet cells and enteroendocrine cells (**Figure S2H-I**). We further analyzed epithelial cell migration dynamics along the crypt-villus axis after a single pulse of BrdU. Upward movement of BrdU^+^ cells from crypts to villi was limited upon *Mex3a* deletion (**Figure S2J**), suggesting impaired epithelial turnover. Taken together, these findings demonstrate that Mex3a maintains the appropriate balance between stem cell proliferation and differentiation for optimal intestinal homeostasis.

To test whether the observed phenotypes were due to a loss of *Mex3a* within the intestinal epithelium, we generated *Villin-Cre*-driven *Mex3a* conditional KO (cKO) mice, in which *Mex3a* was specifically deleted in the intestinal epithelium (**Figure S3A**). Expression levels of *Mex3a* were markedly reduced in the intestinal tissues of cKO mice (**Figure S3B-C**), which also displayed reductions in body weight, crypt depth and number of proliferative cells, as well as excessive differentiation (**Figure S3D-H, Figure EV1A-C**), phenotypes that are generally identical to these in constitutive KO mice. Interestingly, approximately 60% of cKO mice exhibited translucent and air-filled gut tubes, a more robust phenotype than that observed in constitutive KO mice (**Figure S3I**).

**Figure 3:**
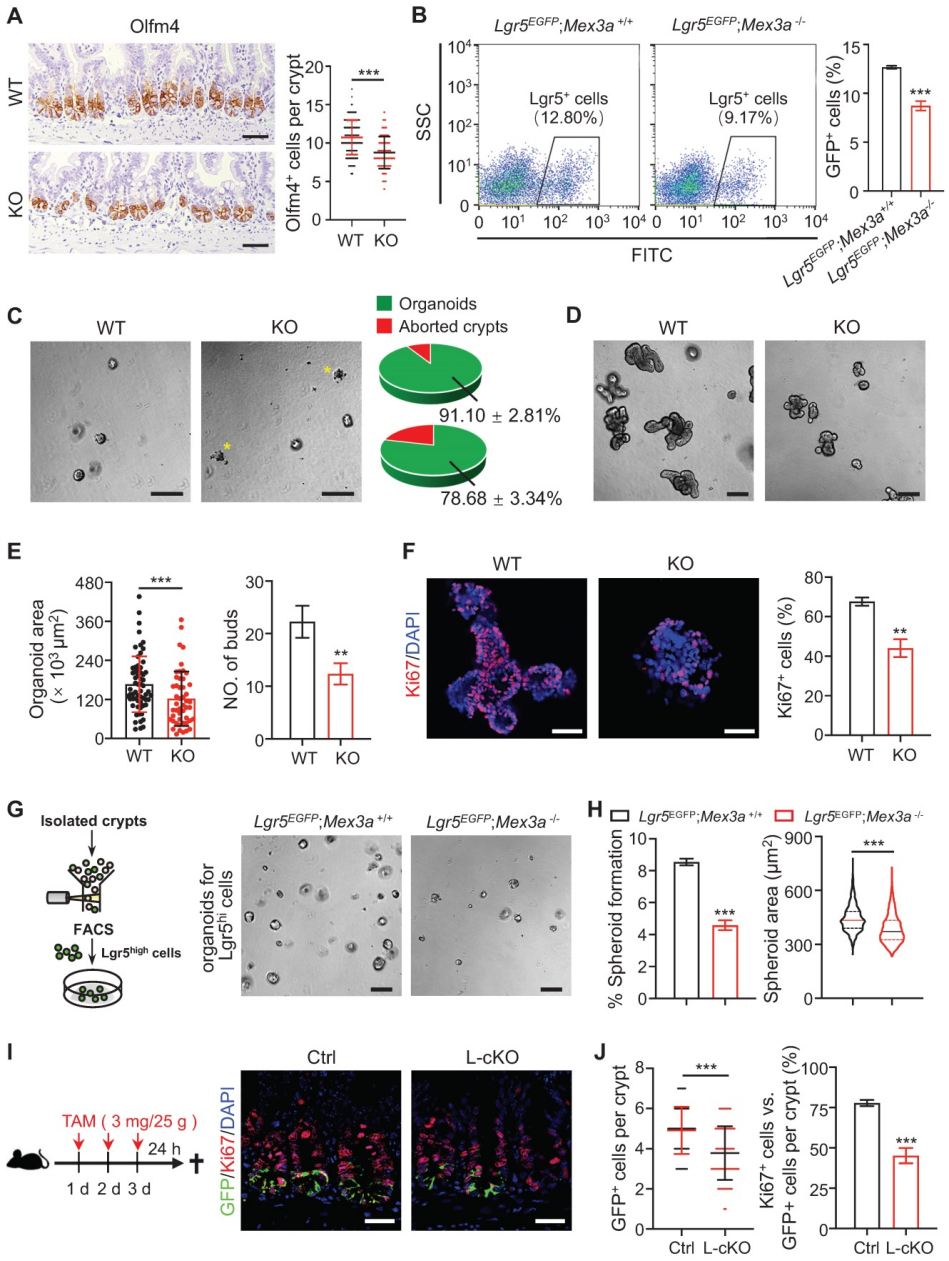
** Deletion of *Mex3a* compromises stemness and proliferative capacity of ISCs. A,** Immunochemistry for Olfm4 in intestinal crypts from wild-type (WT) and KO mice. Numbers of Olfm4^+^ cells per crypt were quantified. WT, n = 162 crypts from 3 mice; KO, n = 139 crypts from 3 mice. Scale bar: 50 μm. **B,** The frequency of Lgr5^+^ ISCs in intestinal crypts from *Lgr5*^EGFP^*;Mex3a*^+/+^ and *Lgr5*^EGFP^*;Mex3a*^-/-^ mice, assayed by flow cytometry. n = 3. **C,** Representative gross images of organoids from WT and KO mice 24 hours after seeding. Growing and aborted organoids were quantified. Yellow asterisks indicate aborted organoid debris. n = 3. Scale bar: 200 μm. **D**-**E,** Intestinal organoid images from WT and KO mice cultured 5 days after passaging (**D**). Organoid area and number of buds were quantified (**E**). n = 3. Scale bar: 200 μm. **F,** Immunofluorescence for Ki67 in intestinal organoids cultured 3 days after seeding. Percentage of Ki67^+^ cells was quantified. n = 3. Scale bar: 50 μm. **G,** Schematic showing isolation and culture of Lgr5^high^ single cells. Representative images of spheroids for sorted Lgr5^high^ single cells from *Lgr5*^EGFP^*;Mex3a*^+/+^ and *Lgr5*^EGFP^*;Mex3a*^-/-^ mice 4 days after seeding. Lgr5^high^ single cells were seeded at the same initial density. n = 2 biologically independent experiments with 3 technical replicates each. Scale bar: 200 μm. **H,** Quantification of the percentage of growing spheroids and spheroid area in panel G. **I,** Schematic for deleting *Mex3a* in Lgr5^+^ cells using *Lgr5^EGFP-CreERT2^;Mex3a^fl/fl^* mice (L-cKO). Intestinal tissues were harvested 24 hours after the last tamoxifen injection. Double immunofluorescence for GFP and Ki67 in ileum from control and L-cKO mice. Scale bar: 25 μm. **J,** Number of GFP^+^ cells per crypt and percentage of Ki67^+^GFP^+^ cells versus GFP^+^ cells per crypt in panel I were quantified. Control, n = 212 crypts, 3 mice; L-cKO, n = 210 crypts, 3 mice. Data are presented as the mean ± SD. *P < 0.05; **P < 0.01; ***P < 0.001.

Considering that *Mex3a* is primarily expressed in Lgr5^high^ ISCs, we next tested the impact of *Mex3a* on ISCs. Olfm4 is a known marker of ISCs 10. The numbers of Olfm4^+^ cells per crypt were significantly reduced in both KO and cKO mice compared with littermate controls (**Figure 3A, S4A**). Consistently, frequency of Lgr5^+^ ISCs significantly decreased in intestinal crypts from *Lgr5^EGFP-CreERT2^;Mex3a^-/-^(Lgr5^EGFP^;Mex3a^-/-^)* mice relative to *Lgr5^EGFP-CreERT2^;Mex3a^+/+^ (Lgr5^EGFP^;Mex3a^+/+^)* mice (**Figure 3B**). Furthermore, mRNA levels of other ISC markers, *Ascl2*, *Lgr5*, *Smoc2* and *Tnfrsf19,* were downregulated in KO mice (**Figure S4B**). In line with this finding, crypts isolated from KO mice displayed lower organoid-initiating capacity than these from littermate controls (**Figure 3C**), and *Mex3a*-deficient organoids grew more slowly and gave rise to fewer buds (**Figure 3D-E, S4C**). Percentage of proliferative cells was markedly reduced in *Mex3a*-deficient organoids (**Figure 3F, S4D**), suggesting impaired proliferative capacity of ISCs. To test the direct effect of *Mex3a* deletion on ISCs, we isolated Lgr5^high^ cells and cultured them in Matrigel at the same initial density. Following four days of culture, Lgr5^high^ cells from *Lgr5^EGFP^;Mex3a*^-/-^ mice had lower spheroid-forming efficiency and gave rise to smaller spheroids than Lgr5^high^ cells from *Lgr5*^EGFP^*;Mex3a*^+/+^ mice (**Figure 3G-H**), suggesting that Mex3a is critical for maintaining ISC stemness. To further verify whether Mex3a directly regulates ISCs *in vivo,* we specifically deleted it in Lgr5^+^ cells using *Lgr5*^EGFP-CreERT2^ mice upon Tamoxifen induction. In agreement with the above observations, inducible deletion of *Mex3a* in Lgr5^+^ cells at adult stage resulted in reduction of Lgr5^+^ ISCs, compromised proliferative capacity of Lgr5^+^ ISCs (**Figure 3I-J, S4E-F**) and excessive differentiation (**Figure EV2A-C**). Thus, Mex3a is critical for maintaining both stemness and proliferative capacity of ISCs and concomitantly prevents excessive differentiation of ISCs.

### MEX3A sustains cancer cell properties

**Figure 4:**
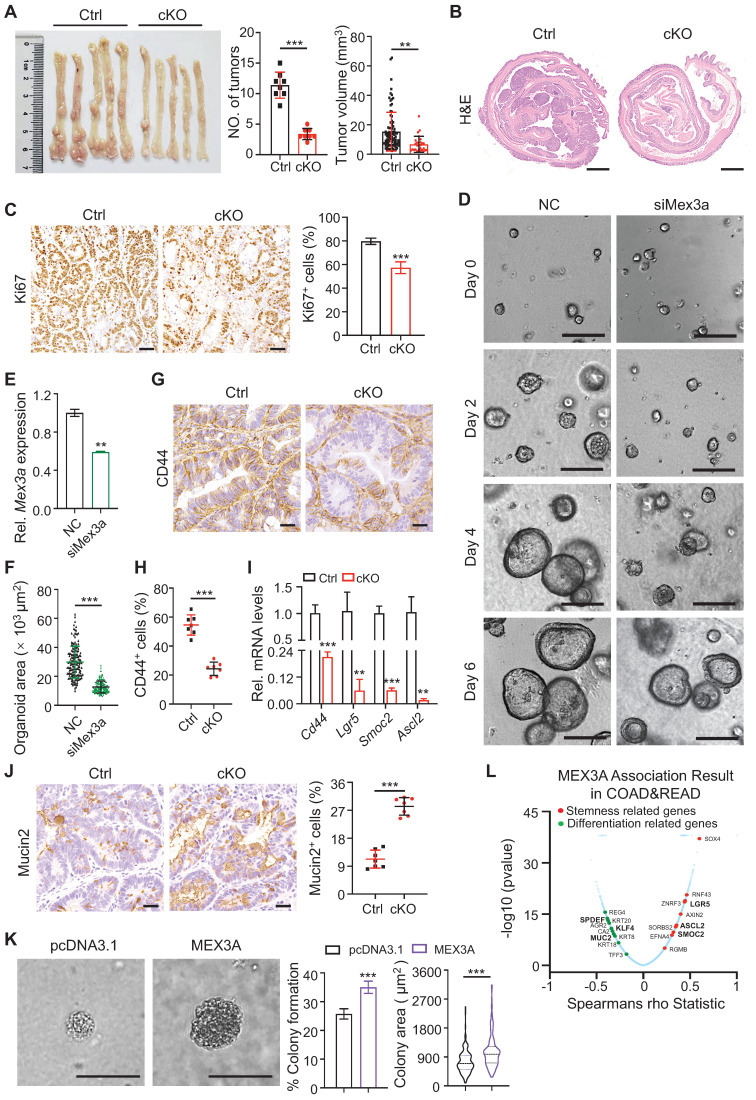
** Deletion of *Mex3a* suppresses tumor growth. A,** Gross images of AOM-DSS mouse colon tumors from control (Ctrl) and *Mex3a* cKO mice. Number of tumors per mouse and tumor volume were quantified. Ctrl: n = 91 tumors from 8 mice. cKO: n = 27 tumors from 8 mice. **B,** Representative histological images of AOM-DSS colon tumors from Ctrl and cKO mice. n = 8. Scale bar: 2 mm. **C,** Immunohistochemistry for Ki67 in AOM-DSS colon tumors from Ctrl and cKO mice. Percentage of Ki67^+^ cells was quantified. n = 7. Scale bar: 50 μm. **D,** Growth of APKS mouse tumor organoids over time. The organoids were transfected with siRNA of *Mex3a* (siMex3a). n = 3. Scale bar: 200 μm. **E,** qRT-PCR analysis of *Mex3a* in mouse tumor organoids after transfection with siMex3a. n = 3. **F,** Quantification of the organoid area in panel D. **G**-**H,** Representative immunohistochemical images for CD44 in AOM-DSS tumors from Ctrl and cKO mice (**G**). Percentage of CD44^+^ cells was quantified (**H**). n = 7. Scale bar: 50 μm. **I,** qRT-PCR for cancer stem cell markers *Cd44*, *Lgr5*, *Smoc2* and *Ascl2* in AOM-DSS tumors from Ctrl and cKO mice. n = 3.** J,** Immunohistochemistry for Mucin2 in AOM-DSS tumors from Ctrl and cKO mice. Percentage of Mucin2^+^ cells was quantified. n = 7. Scale bar: 50 μm.** K,** Anchorage-independent growth of HCT116 cells transfected with pcDNA3.1 empty vector or pcDNA3.1-MEX3A plasmids. Colony-forming percentage and colony area were quantified. Colonies were grown for over 3 weeks. n = 3. Scale bar: 50 μm. **L,** Volcano plot of significantly correlated genes with *MEX3A* in COAD and READ. Stemness related genes are marked in red and differentiation related genes are marked in green. Data are presented as the mean ± SD. *P < 0.05; **P < 0.01; ***P < 0.001.

Considering that Mex3a promotes ISC stemness and become overexpressed in CRC, we hypothesized that MEX3A contributes to cancer progression. First, we examined its *in vivo* role in AOM-DSS-induced tumor model, that recapitulates inflammation-driven colorectal adenocarcinoma 29. We found that *Mex3a* deficiency led to a remarkable reduction both in tumor size and their numbers (**Figure 4A**), while no significant differences in body weight changes between control and cKO mice were observed during tumor development (**Figure S5A**). Histological analysis revealed marked reduction in tumor lesions upon *Mex3a* deficiency (**Figure 4B**). In agreement with this observation, the percentage of proliferative cells was significantly lower in tumors from cKO mice than control mice (**Figure 4C, Figure EV3A-B**). Further supporting the pro-oncogenic function of MEX3A, *in vitro* data showed that its overexpression promotes proliferation in HCT116 human colon cancer cell line (**Figure S5B-D**), and shifted relative cell cycle phase distribution toward S phase (**Figure S5E**). Conversely, suppression of *MEX3A* in HCT116 cells with siRNA abrogated their proliferation and induced cell cycle arrest (**Figure S5F-I**). Furthermore, using CRISPR/Cas9 system we generated the so-called APKS mouse tumor organoid, harboring mutations in *Apc*, *Tp53*, *Kras* and *Smad4* 30, 31 (**Figure S6A-D**) - the driver genes of oncogenic transformation in CRC. We show that siRNA-mediated* Mex3a* knockdown in such organoids significantly suppresses their growth (**Figure 4D-F, Figure EV3C-E**). Together, these findings demonstrate that MEX3A promotes tumor growth by enhancing the proliferative capacity of CRC cells.

Next, we investigated whether MEX3A regulates stemness of colon tumor cells. First, we examined the expression of cancer stem cell marker gene CD44 32, in AOM-DSS-induced tumor model. Frequency of CD44^+^ cells (**Figure 4G-H**) and expression levels of *Cd44* and other cancer stem cell markers *Ascl2*, *Lgr5* and *Smoc2* were markedly reduced in *Mex3a*-deficient tumors (**Figure 4I**). Conversely, frequency of differentiated Mucin2^+^ cells was elevated upon *Mex3a* deficiency (**Figure 4J**). It suggests that *Mex3a* functions as an oncogene in retaining stemness and rapidly-dividing status of tumor cells. Furthermore, the oncogenic functions of Mex3a were further validated when *Mex3a* was deleted in *Vil-Cre;APC^fl/+^*mice (**Figure S7A-H**), in which *APC* mutation-driven tumor mouse model is relevant to human familial CRC.

To further examine the direct impact of MEX3A on cancer cell stemness, we measured anchorage-independent growth of HCT116 cells upon *MEX3A* overexpression. We found that both the frequency and the size of colonies growing in an anchorage-independent manner, which is a hallmark of oncogenic transformation 33, markedly increased in response to *MEX3A* (**Figure 4K**), suggesting enhanced stemness and proliferative capacity of cancer stem cells. Furthermore, we induced spheroid formation in HCT116 cells to enrich CSCs, in which stemness-related marker genes were upregulated (**Figure S8A-C**). *MEX3A* knockdown strongly inhibited formation and growth of HCT116 tumor spheroid (diameter ≥ 50 μm), conversely *MEX3A* overexpression promoted it (**Figure S8D-G**).

Accordingly, the percentage of CD44^+^ cells and SOX2^+^ cells reduced in tumor spheroids upon *MEX3A* knockdown, while they increased upon *MEX3A* overexpression (**Figure S8H-J**). Further supporting this notion, correlation analysis between *MEX3A* and the altered genes in the TCGA COAD (colon adenocarcinoma) cohort (n = 391) and READ (rectal adenocarcinoma) cohort (n = 152) cohort using the *Function* module of LinkedOmics 34 revealed that *MEX3A* positively correlates with stemness-related genes, and negatively correlates with differentiation-related genes (**Figure 4L**). Taken together, these data suggest that *MEX3A* functions as an oncogene in retaining stemness and rapidly-dividing status of tumor cells.

### MEX3A enhances cancer cell resistance to radiation

**Figure 5:**
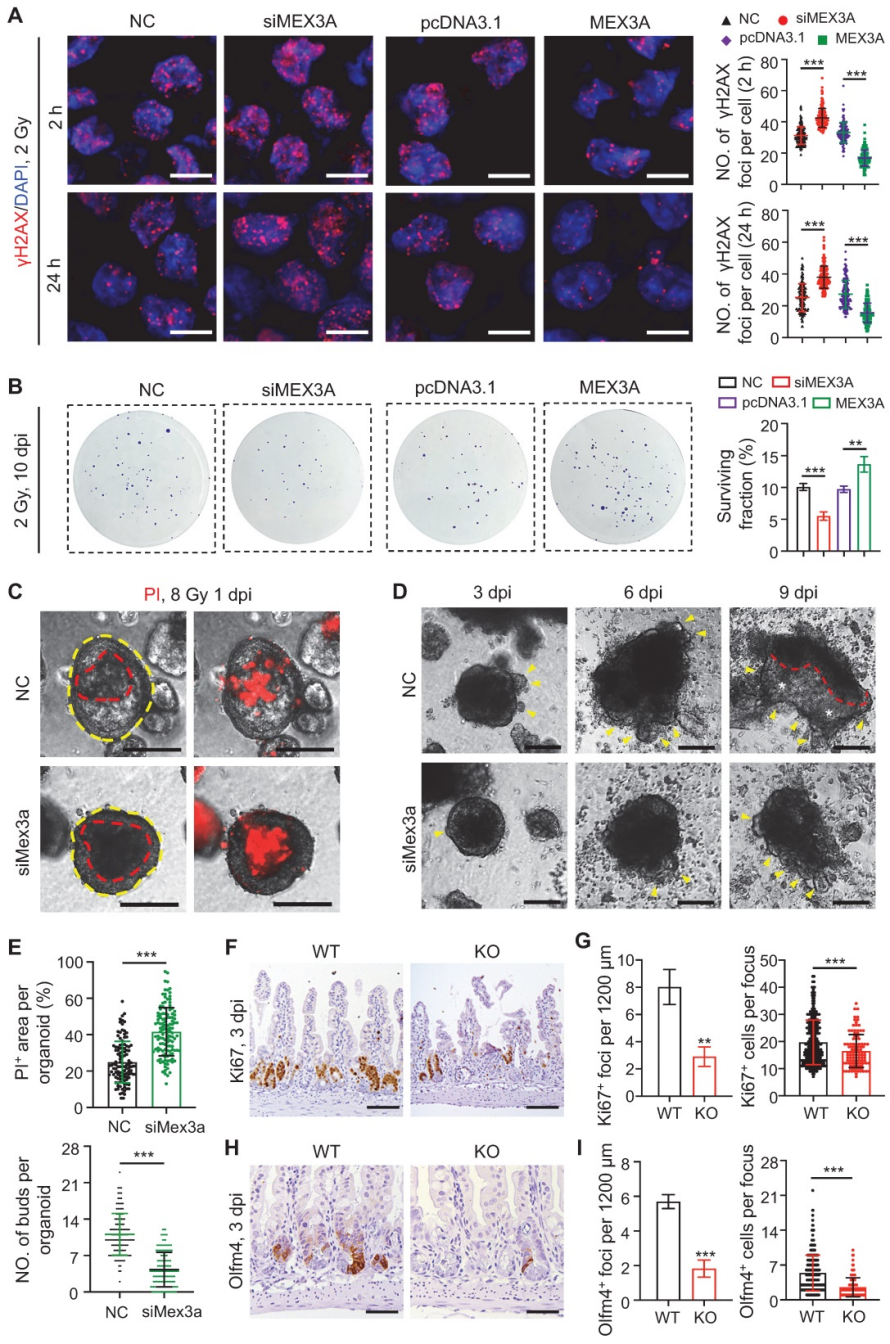
** Silencing *Mex3a* sensitizes cancer cells to irradiation. A,** Immunofluorescence for γH2AX in HCT116 transfected with siMex3a or pcDNA3.1-Mex3a plasmids 2 hours and 24 hours post 2 Gy γ-radiation. Numbers of γH2AX^+^ foci per cell were quantified. n = 3. Scale bar: 25 μm. **B,** Clonogenic assay of HCT116 cells transfected with siMex3a or pcDNA3.1-Mex3a plasmids following radiation. Surviving fractions were calculated. n = 3. **C,** PI staining of APKS mouse tumor organoids transfected with siMex3a 24 hours after 8 Gy γ-radiation. Red line indicates the area of cell death. n = 4. Scale bar: 200 μm. **D,** Representative gross images of mouse tumor organoids transfected with siMex3a at indicated timepoints post 8 Gy γ-radiation. Yellow arrowheads indicate newly generating organoid buds. Newly generating zones are indicated with white asterisks. n = 3. Scale bar: 200 μm.** E,** Quantification of PI^+^ area per organoid in panel C and numbers of buds per organoid in panel D. **F,** Immunohistochemistry for Ki67 in ileum from wild-type (WT) and KO mice 3 days postirradiation. Scale bar: 100 μm. **G,** Quantification of Ki67^+^ regenerative foci per 1200 μm and number of Ki67^+^ cells per regenerative focus in panel F. n = 3. **H,** Immunohistochemistry for Olfm4 in ileum from WT and KO mice 3 days postirradiation. Scale bar: 50 μm. **I,** Quantification of Olfm4^+^ foci per 1200 μm and numbers of Olfm4^+^ cells per focus in panel H. n = 3. Data are presented as the mean ± SD. *P < 0.05; **P < 0.01; ***P < 0.001.

Next we asked if MEX3A contributes to radiation therapy resistance, a property attributed to cancer stem cells 8. When HCT116 cells were exposed to 2 Gy of irradiation, *MEX3A* knockdown and overexpression resulted in increase and decrease in DNA damage marker γH2AX^+^ foci, respectively (**Figure 5A**). Consistently, *MEX3A* knockdown and overexpression resulted in decrease and increase in colony formation by 2 Gy-treated HCT116 cells, respectively (**Figure 5B**). Furthermore, *Mex3a* knockdown promoted cell death in irradiated APKS mouse tumor organoids (**Figure 5C-E**) and led to their slower recovery as compared to control (**Figure 5D-E**), suggesting that *Mex3a* silencing sensitizes tumor cells to radiation-induced DNA damage.

To test radio-resistant role of Mex3a *in vivo*, we exposed control and *Mex3a* KO mice to 12 Gy of irradiation. Mutant mice were more susceptible to irradiation and showed reduced survival rate (**Figure S9A**). Frequency of regenerative foci and numbers of proliferative cells per focus at three days post-irradiation were both markedly lower in KO mice relative to littermate controls (**Figure 5F-G, S9B-C**). Both frequency of Olfm4^+^ foci and numbers of Olfm4^+^ stem/progenitor cells per focus were also reduced in mutants at the same time point (**Figure 5H-I**). Relative to control, KO mice showed more DNA damage response (DDR) marker-positive epithelial cells in intestinal crypts at 24 hours post-irradiation, suggesting more severe DNA damage (**Figure S9D-F**). Taken together, these findings indicate that Mex3a enhances cellular resistance to radiation *in vivo*.

### MEX3A activates WNT signaling pathway

**Figure 6:**
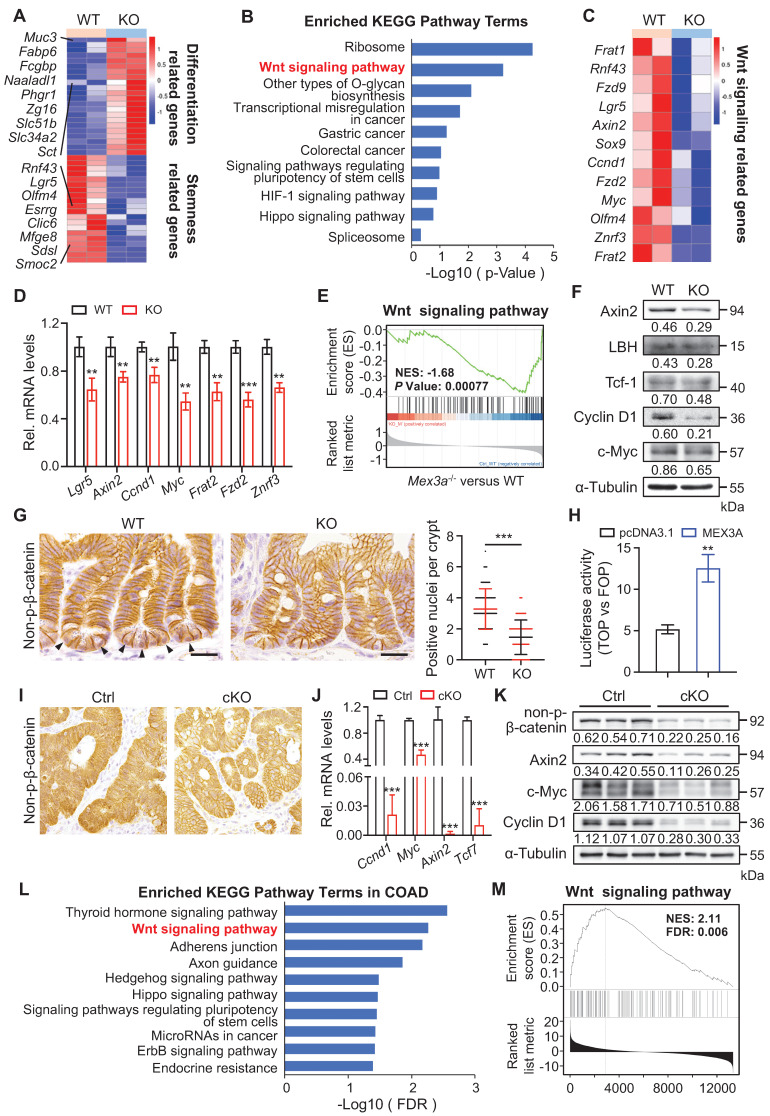
***Mex3a* deletion suppresses WNT signaling activity. A,** Heatmap depicting the expression profiles of stemness related genes and differentiation related genes in transcriptome profiles of wild-type (WT) and KO mice. **B,** KEGG pathway analysis of downregulated genes in transcriptome profiles from WT and KO mice. n = 4 biological replicates. **C,** Heatmap showing altered WNT-related genes in WT and KO mice. **D,** qRT-PCR analysis validates altered WNT-related genes in WT and KO mice. n = 3. **E,** Gene set enrichment analysis (GSEA) of WNT signaling pathway genes in transcriptome profiles of WT and KO mice. **F,** Western blotting for WNT target genes c-Myc, Cyclin D1, Tcf-1, LBH and Axin2 in intestinal crypts from WT and KO mice. α-Tubulin was used as a loading control. **G,** Representative immunohistochemical images of non-p-β-catenin in ileum from WT and KO mice. Arrowheads point to non-p-nuclei. Numbers of nuclear β-catenin^+^ cells per crypt were quantified. WT, n = 245 crypts from 3 mice; KO, n = 253 crypts from 3 mice. Scale bar: 25 μm. **H,** Luciferase activity of TOPflash versus FOPflash in HCT116 cells treated with pcDNA3.1 empty vector and MEX3A plasmids. n = 3. **I,** Immunohistochemistry for non-p-β-catenin in AOM-DSS colon tumors from control (Ctrl) and cKO mice. n = 7. Scale bar: 25 μm.** J,** qRT-PCR analysis of WNT target genes in AOM-DSS tumors from Ctrl and cKO mice. n = 3. **K,** Western blotting for Cyclin D1, c-Myc, Axin2 and non-p-β-catenin in AOM-DSS tumors from Ctrl and cKO mice. α-Tubulin was used as a loading control. **L,** KEGG pathway analysis of genes positively correlated with *MEX3A* in COAD. **M,** Gene set enrichment analysis of WNT signaling pathway in genes positively correlated with *MEX3A* in COAD. Data are presented as the mean ± SD. *P < 0.05; **P < 0.01; ***P < 0.001.

To gain insight into the molecular mechanism of how MEX3A regulates stemness and proliferative capacity of both ISCs and cancer cells, we performed genome-wide transcriptome analysis on intestinal crypt cells from control (n = 4) and KO (n = 4) mice. Differentially expressed genes were defined as those with *P* < 0.05. In total, 511 downregulated and 1112 upregulated genes were identified in mutants (**Figure S10A**). In line with the observed phenotypes, differentiation-related genes were upregulated in KO mice, while stemness-related genes were downregulated (**Figure 6A**). KEGG analysis revealed that pathways most enriched among downregulated genes include ribosome, WNT signaling, colorectal cancer and signaling pathways regulating pluripotency (**Figure 6B**). Importantly, WNT signaling is a major driver of both ISC renewal and oncogenic transformation upon CRC 35. Further analysis revealed that a number of WNT-responsive genes, including *Axin2*, *Fzd2/9*, *Sox9*, *Ccnd1* and *Myc,* were downregulated in *Mex3a*-depleted crypt cells (**Figure 6C**) - a finding confirmed by qRT-PCR (**Figure 6D**). Gene set enrichment analysis (GSEA) further showed positive relationship between Mex3a and WNT activity (**Figure 6E**). On these basis, we proposed that Mex3a may upregulate WNT signaling.

Supporting this possibility, WNT targets Axin2, LBH, c-Myc, Cyclin D1 and Tcf-1 were significantly downregulated at the protein level in KO intestinal crypts (**Figure 6F**). There were also substantially fewer nuclear β-catenin^+^ cells in intestinal crypts from both KO and cKO mice than in these from littermate controls (**Figure 6G, S10B**). Reduction in nuclear β-catenin^+^ cells became more pronounced in regenerative foci from KO mice at three days post-irradiation (**Figure S10C**). Furthermore, TOPflash/FOPflash reporter assay showed that *MEX3A* induction enhanced WNT signaling activity (**Figure 6H**). Thus, we posit that MEX3A activates WNT pathway.

We then examined MEX3A/WNT connection in colon tumors. Responding to *MEX3A* inhibition, WNT target genes *CCND1*, *TCF7* and *AXIN2* became markedly reduced in HCT116 cells *in vitro* both at RNA and protein levels (**Figure S10D-E**). Considering that HCT116 cells are heterozygous for β-catenin gain-of-function mutation at the Gsk3β target site S45 (Ctnnb1^+/S45mt^) 36, we proposed that MEX3A activates WNT downstream of Gsk3β. *In vivo,* we found a robust decrease in WNT activity in AOM-DSS-induced colon tumors and conditional *Apc*-mutant intestinal tumors from cKO mice (**Figure 6I-K, S10F-G**). Moreover, upon LinkedOmics analysis of transcriptome profiles of 379 colon adenocarcinomas (COAD) in TCGA, we identified that 6237 genes significantly and positively correlate with MEX3A. Likewise, KEGG analysis ranked WNT signaling pathway second among the enriched terms (**Figure 6L**), while GSEA assay showed positive relationship between MEX3A and WNT activity in colon adenocarcinoma (**Figure 6M**). Concomitantly, analysis of TCGA data showed positive correlation between MEX3A and WNT target genes *AXIN2*, *MYC* and *TCF7* (**Figure S10H-J**). Collectivelly, these findings demonstrate that MEX3A activates WNT signaling in colon cancer.

### MEX3A directly targets KLF4 for WNT activation

**Figure 7:**
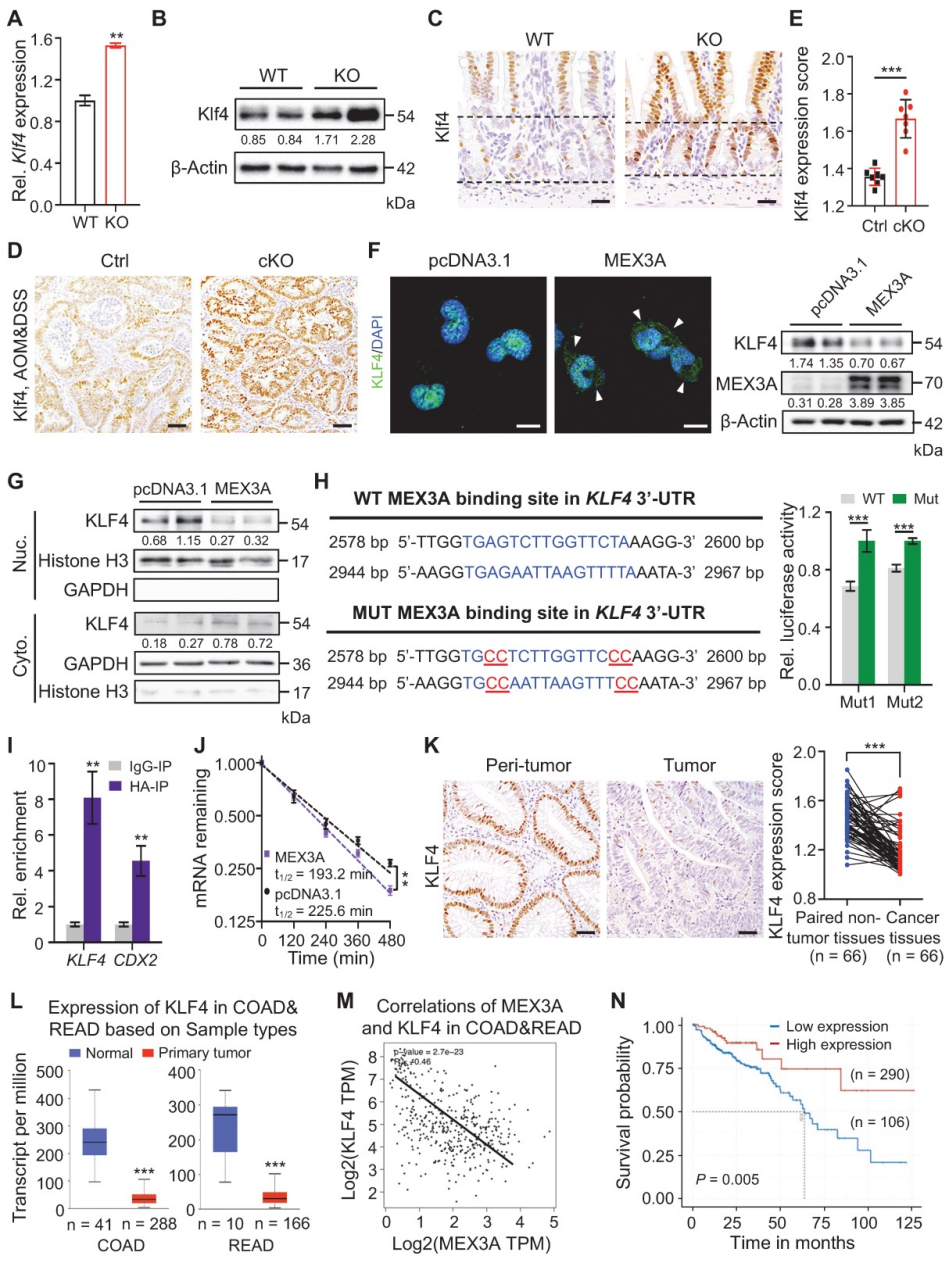
** MEX3A directly suppresses *KLF4* to activate WNT signaling. A**-**B,** qRT-PCR (**A**, n = 3) and Western blotting (**B**) for Klf4 in ileum tissues from wild-type (WT) and KO mice. α-Tubulin was used as loading control. **C,** Representative immunohistochemical images of Klf4 in intestinal crypts from WT and KO. The dashed lines indicate villus-crypt junction and base of the crypt. n = 3. Scale bar: 25 μm. **D-E,** Immunohistochemistry for Klf4 in AOM-DSS colon tumors from control (Ctrl) and cKO mice (**D**). Scores of Klf4 expression levels were quantified (**E**). n = 3. Scale bar: 50 μm. **F,** Immunofluorescence for KLF4 and Western blotting for MEX3A and KLF4 in NCM460 cells treated with empty vector or MEX3A-overexpressing plasmids. Scale bar: 25 μm. β-Actin was used as a loading control. Arrowheads indicate KLF4 signal in cytoplasm. **G,** Western blotting for KLF4 in nuclear and cytoplasmic proteins isolated from NCM460 cells treated with empty vector or MEX3A-overexpressing plasmids. Histone H3 and GAPDH were used as positive control for nuclear and cytoplasmic proteins, respectively. **H,** Ratio of luciferase activity in MEX3A-overexpressing versus normal HEK293T cells transfected with luciferase reporter vector containing a *KLF4* 3'-UTR fragment with WT sequence or mutations (Mut1 or Mut2) in MEX3A binding sites. n = 3. **I,** Crosslinking-Immunoprecipitation (CLIP)-PCR assay for *KLF4* and *CDX2* upon the anti-HA (MEX3A-HA) antibody immunoprecipitates. n = 3 technical replicates. **J,** The *KLF4* mRNA decays curve in HCT116 cells upon *MEX3A* overexpression. n = 3. **K,** Immunohistochemistry for KLF4 in human colorectal tumors and paired peritumor samples. Scores of KLF4 expression levels were quantified. Scale bar: 50 μm. **L,** Box plots of KLF4 expression in normal human colorectum and CRC tissues based on TCGA database. n, number of patient samples. **M,** Spearman correlation analysis of MEX3A and KLF4 (*P* < 0.001; *R* = - 0.46) in human CRC based on TCGA database. **N,** Kaplan-Meier survival curve of 396 CRC patients. *P* = 0.005. Data are presented as the mean ± SD. *P < 0.05; **P < 0.01; ***P < 0.001.

Next, we sought to identify putative direct target gene(s) of MEX3A by profiling sequences of differentially expressed genes for its recognition element: (A/G/U)(G/U)AGN_(0-8)_U(U/A/C)UA 37. Of the candidate genes, WNT inhibitor *Klf4* 38, 39 was of particular interest. It is upregulated in KO mice, which we validated on qRT-PCR and Western blot (**Figure 7A-B**). Furthermore, deletion of *Mex3a* resulted in Klf4 upregulation in intestinal crypts where Mex3a is normally present (**Figure 7C**). Klf4 protein was barely detectable in normal regenerative foci where *Mex3a* is highly expressed, but became markedly elevated in *Mex3a*-deficient regenerative foci (**Figure S11A**). Compared to control, expression levels of Klf4 significantly increased in AOM-DSS-induced colon tumors from cKO mice (**Figure 7D-E**), while *MEX3A* overexpression in NCM460 cells downregulated KLF4 protein and restricted its nuclear localization (**Figure 7F-G**). These data suggest direct impact of MEX3A on KLF4.

Two putative MEX3A binding sites were identified in the 3'UTR of *Klf4*/*KLF4* which are conserved between mouse and human (**Figure 7H, S11B**). RNA-Protein Interaction Prediction (RPI-Seq) analysis showed that predicted probability of MEX3A interacting with *KLF4* was 0.9 with the random forest (RF) classifier and 0.98 with the support vector machine (SVM) classifier -both high values (**Figure S11C**). To test whether MEX3A directly suppresses 3'-UTR activity of *KLF4*, we constructed luciferase reporter vector containing either wild-type or mutated MEX3A binding sites. Mutation of binding sites 1 or 2 significantly blocked the MEX3A overexpression-mediated suppression of luciferase activity in HEK293T cells (**Figure 7H**). Next, RNA crosslinking immunoprecipitation (CLIP)-qPCR assay revealed enrichment for *KLF4* in MEX3A-immunoprecipitate relative to control IgG immunoprecipitate (IgG-IP) in HCT116 cells (**Figure 7I**). Accuracy of the assay was validated by studying known MEX3A target gene *CDX2*. Additionally, *MEX3A* overexpression accelerated *KLF4* mRNA decay (**Figure 7J**). Together, these findings demonstrate that MEX3A directly suppresses *KLF4* expression by de-stabilizing its mRNA.

We then sought to examine conservation of MEX3A / KLF4 relationship in human colorectal cancer. Immunohistochemical analysis showed that KLF4 is significantly downregulated in human colorectal tumors relative to peritumor tissues, exhibiting negative correlation with MEX3A (**Figure 7K, S11D-E**). Downregulation of KLF4 in colorectum tumors and negative correlation with MEX3A were further confirmed by analysing TCGA data (**Figure 7L-M**). Importantly, in contrast with *MEX3A*, low *KLF4* expression associates with poor survival (**Figure 7N**). Considering that KLF4 is a WNT inhibitor 38, 39, we concluded that MEX3A directly suppresses KLF4 to activate WNT signaling both in ISCs and CRC. Because KLF4 is an established regulator of terminal differentiation of intestinal epithelial cells 28, 40, we asked whether it also responds to intestinal differentiation-inducing BMP signaling 5. We treated 3D cultures of Caco-2 colon cancer cells with BMP4 protein and it significantly upregulated *KLF4* (**Figure S11F**). Thus, we proposed that MEX3A-KLF4 axis coordinates WNT and BMP signaling activities, helping to balance self-renewal and differentiation in ISCs and CSCs.

### Targeting MEX3A and KLF4 offers potent differentiation therapeutic strategy for CRC

**Figure 8:**
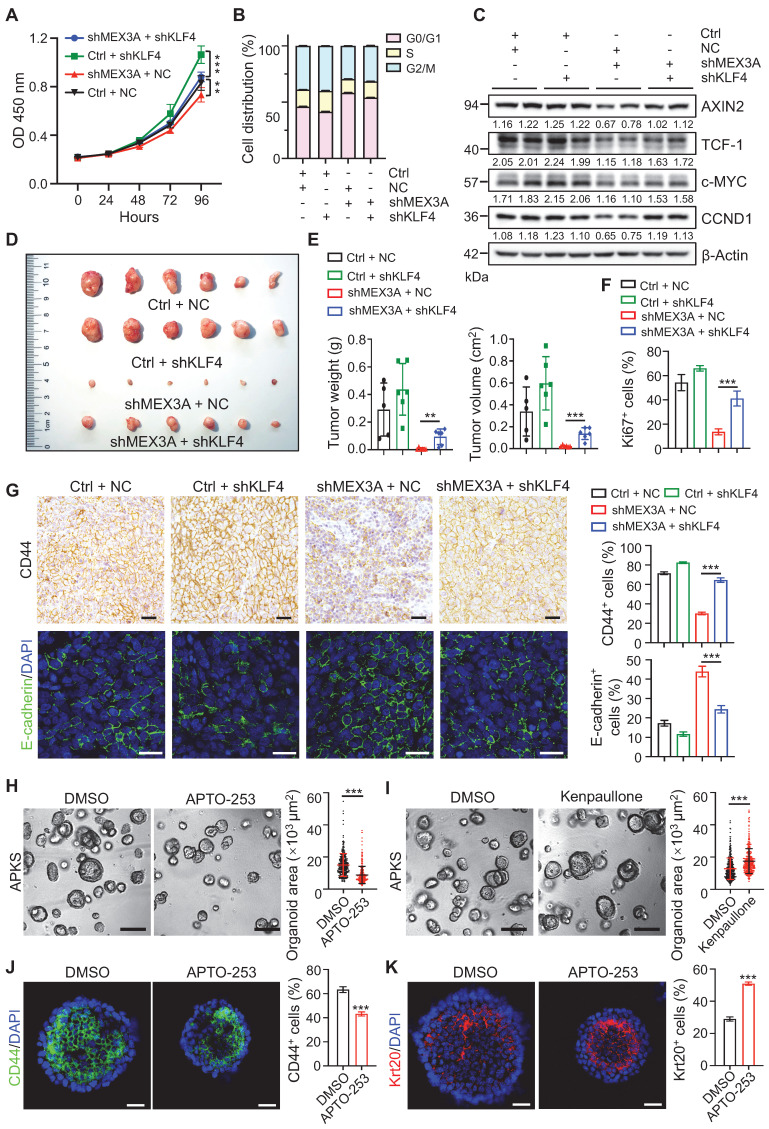
** KLF4 mediates promoting effects of MEX3A on tumor growth and stemness. A,** Growth curve of HCT116 cells transfected with shMEX3A and/or shKLF4 over time. n = 6. **B,** Cell cycle analysis with flow cytometry for HCT116 cells transfected with shMEX3A and/or shKLF4. n = 3. **C,** Western blotting for CCND1, c-MYC, TCF-1 and AXIN2 in HCT116 cells treated with shMEX3A and/or shKLF4. β-Actin was used as a loading control. **D,** Gross images of xenografted tumors 3 weeks after transplantation with shMEX3A-and/or shKLF4-transfected HCT116 cells. **E,** Quantification for tumor weight and volume shown in panel D. n = 6. **F,** Quantification of percentages of Ki67^+^ cells in xenografted tumors shown in Figure S12H. n = 6. **G,** Immunostaining for CD44 and E-cadherin in xenografted tumors from HCT116 cells transfected with shMEX3A and/or shKLF4. The percentages of CD44^+^ cells and E-cadherin^+^ cells were quantified. n = 6. Scale bar: 50 μm. **H**-**I,** Growth of mouse tumor organoids after KLF4 activation (**H**) or inhibition (**I**). Organoids were grown for 48 hours and then treated with KLF4 agonist APTO-253 or KLF4 antagonist Kenpaullone for 72 hours. DMSO was used as solvent control. Organoid area was quantified. n = 3 technical replicates. Scale bar: 200 μm. **J**-**K,** Immunofluorescence for CD44 (**J**) and Krt20 (**K**) in mouse tumor organoids upon Klf4 activation. The percentages of CD44^+^ cells and Krt20^+^ cells were quantified. n = 3. Scale bar: 25 μm. Data are presented as the mean ± SD. *P < 0.05; **P < 0.01; ***P < 0.001.

To test whether KLF4 mediates the effects of *MEX3A* deficiency, we asked if *KLF4* suppression rescues *MEX3A* deletion phenotypes. First, *in vitro* assays showed that *KLF4* knockdown abrogated inhibitory effects of *MEX3A* suppression on cell growth and cell cycle progression (**Figure 8A-B, S12A-C**). Correspondingly, *MEX3A* suppression-induced downregulation of WNT target genes was also rescued by *KLF4* knockdown (**Figure 8C, S12D**). In contrast, *KLF4* overexpression abolished MEX3A-induced stimulation of cell proliferation (**Figure S12E**), accompanied by reduced WNT activity (**Figure S12F-G**). Importantly, tumor xenograft assay showed that *MEX3A* knockdown significantly suppresses tumor growth, while *KLF4* knockdown rescues *MEX3A* inhibition-induced suppression of tumor growth (**Figure 8D-E, Figure EV4A-C**). Notably, *MEX3A* inhibition not only suppresses proliferative capacity of tumor cells (**Figure 8F, S12H-I**), but also renders tumor cell-state conversion from stemness to differentiation, evidenced by reduction of CD44 and upregulation of E-cadherin (**Figure 8G**). Conversely, *KLF4* knockdown rescues *MEX3A* inhibition-induced effects. These observations imply that inhibition of MEX3A or upregulation of KLF4 might represent new targets for cancer differentiation therapy, a promising strategy for cancer treatment.

As a proof-of-principle, we treated APKS mouse tumor organoids with KLF4 agonist APTO-253, and it significantly suppressed tumor organoid growth. Conversely, KLF4 inhibitor Kenpaullone promoted tumor organoid growth (**Figure 8H-I, S12J-K**). In agreement with the compromised organoid growth, APTO-253 treatment converted undifferentiated organoid cells to more differentiated cells (**Figure 8J-K**). These findings suggest that KLF4 agonist can suppress cancer development, which might represent a valid therapeutic compound for CRC patients with high levels of MEX3A.

## Discussion

Our work demonstrates that E2F3-induced MEX3A directly suppresses terminal differentiation regulator KLF4 to activate WNT signaling, that in turn sustains cancer cells in rapidly-dividing and undifferentiated state (**Figure S13**). That inhibition of *MEX3A* suppressed proliferative capacity of tumor cells and forced their differentiation, while *MEX3A* overexpression promoted it, highlighting its pro-tumorigenic role in CRC. In line with this, *MEX3A* upstream regulator E2F3 also functions as an oncogenic transcription factor in a variety of cancers 41-43, and high E2F3 levels confer poor overall survival and disease-free survival in patients with colon cancer 27. Our study also demonstrates that MEX3A directly suppresses terminal differentiation regulator *KLF4*. Supporting oncogenic function of MEX3A, *KLF4* is downregulated in CRC and functions as tumor suppressor 44 and it inhibits WNT signaling activity in HCT116 colon cancer cells by competitively interacting with β-catenin 38, 39. Notably, our findings and previous reports have identified KLF4 as a downstream effector of BMP pathway, an important regulator of terminal differentiation in intestinal epithelial cells 45, 46. This suggest that MEX3A activity antagonizes BMP-KLF4 signaling-induced differentiation process and maintains WNT signaling to retain CSC/ISCs in undifferentiated and rapidly-dividing status. Considering that WNT activation is an essential driver of CRC 35, it appears that E2F3-MEX3A-KLF4 cascade functions as a molecular switch in balancing opposing WNT and BMP effects on CSC self-renewal and differentiation.

Cancer differentiation therapy has long been suggested as a promising strategy for tumor treatment with low side effect profile 7. This strategy aims to convert undifferentiated highly malignant cancer cells into differentiated cells with low tumorigenic potential 47. Considering the uncovered importance of E2F3-MEX3A-KLF4 cascade on self-renewal and differentiation by CSCs, it represents a new molecular target for CRC differentiation therapy. Supporting this possibility is our data with KLF4 agonist APTO-253 that suppresses colon tumor organoid growth by promoting differentiation. This merits future work to identify small molecule inhibitor(s) for MEX3A or new agonists for KLF4 and to test their potential for treating CRC.

Radiotherapy is broadly applied in oncologic treatment, but many tumors respond poorly to it because of radioresistance of CSCs and tumor recurrence 48, 49. Our study reveals that MEX3A enhances radioresistance and injury-induced regeneration of cancer cells. This notion corroborates a previous study that Mex3a-expressing intestinal cells are resistant to radiation 18. Supporting the role of MEX3A in radioresistance of colorectal cancer cells, it has been reported that the upstream regulator E2F3 functions on DNA damage response 50. In agreement with the importance of MEX3A in radioresistance, the reduction of its downstream effector KLF4 enhances radiosensitivity of HCT116 cells 51. Taken together, these findings indicated that the E2F3-MEX3A-KLF4 cascade is important for the radioresistance of cancer cells.

It is also important that Mex3a is critical for maintaining ISC stemness and proliferation under physiological conditions. Interestingly, Pereira *et al.* recently reported that Mex3a regulates Lgr5^+^ stem cell maintenance in developing intestine by downregulating PPARγ activity 19. While exciting, the molecular mechanism underlying PPARγ regulation remains unclear. In this regard, it is worth noting that PPARγ and WNT signaling exhibit reciprocal, opposing crosstalk in many biological contexts 52-54 and, in particular, WNT signaling can downregulate *PPARγ* expression 53. Our work demonstrates that Mex3a activates WNT in ISCs by directly suppressing *Klf4.* The intestinal phenotypes of *Mex3a*-deficient mice recapitulate these induced by loss of WNT function 55, 56. Thus, it is likely that Mex3a indirectly suppresses PPARγ activity via Klf4-mediated WNT signaling activation in ISCs.

## Supplementary Material

Supplementary figures and tables.

## Abbreviations

ISC: Intestinal stem cell
CRC: Colorectal cancer
CSC: Cancer stem cell
APC: Adenomatous polyposis coli
RBP: RNA-binding protein
TCGA: The Cancer Genome Atlas
AOM-DSS: Azoxymethane-dextran sulfate sodium
DDR: DNA damage response
COAD: Colon adenocarcinoma
GSEA: Gene set enrichment analysis
RPI-Seq: RNA-protein interaction prediction
RF: Random forest
SVM: Support vector machine
RNA-seq: Transcriptomic RNA sequencing
CLIP: RNA crosslinking immunoprecipitation
