# Supplementary material for: Identifying the E2F3-MEX3A-KLF4 signaling axis that sustains cancer cells in undifferentiated and proliferative state
Source: Theranostics. 2022 Sep 25;12(16):6865–82. doi: 10.7150/thno.76619 (PMC9576623; doi:10.7150/thno.76619)
Supplement: Supplementary file 1 — Supplementary figures and tables. [file thnov12p6865s1.pdf]

Supplementary Materials for

Identifying the E2F3-MEX3A-KLF4 signaling axis that sustains cancer cells in undifferentiated and proliferative state

Xu Yang[1,2†], Guilin Li[1†], Yuhua Tian[3], Xin Wang[3], Jiuzhi Xu[4], Ruiqi Liu[1], Min Deng[1], Chunlei Shao[1], Yuwei Pan[1], Xi Wu[1], Mengzhen Li[1], Chaowei Zhang[5], Rui Liu[6], Jun Qin[7], Chen Zhang[8], Zhanju Liu[9], Xin Wu[10], Maksim V. Plikus[11], Christopher J. Lengner[3], Zongheng Zheng[5]*, Cong Lv[4]* and Zhengquan Yu[1]*

Correspondence to: zyu@cau.edu.cn (Z.Y.); lvc@cau.edu.cn (C.L.); zhengzh@mail.sysu.edu.cn (Z. Z.)

This PDF file includes:

Supplementary Materials and Methods
Supplementary Figures S1 to S13
Expanded View Figures EV1 to EV4
Supplementary Tables S1 to S4

Supplementary Materials and Methods

In situ hybridization

Small intestine tissues from 8-week-old mice were fixed with 4% PFA at room temperature (RT) for 24 h and embedded in paraffin. The embedded tissues were cut into 5 μm sections. The sample treatment and signal detection were performed according to the corresponding manufacturer's instructions (322452 USM and 322360 USM). An RNAscope 2.5 HD detection kit (RED) and Mm-Mex3a probe (ACD, 318531) were used in this experiment.

Histology, immunohistochemistry and immunofluorescence

For histological analysis, 4% PFA-fixed and paraffin-embedded intestinal tissues were cut into 5 μm sections. The sections were deparaffinized with xylene followed by treatment with serial dilutions of ethanol. Then, the sections were stained with hematoxylin and eosin (H&E, Sigma). Periodic acid–Schiff (PAS)-alkaline phosphatase staining was performed using standard methods. For immunohistochemistry, heat-mediated antigen retrieval was performed using 0.01 M citrate buffer (pH 6.0) or 1 mM EDTA (pH 8.0) for 20 min in a microwave. After cooling at room temperature, the sections were immersed in 3% H_2O_2 for 10 min, or permeabilized with 1% Triton X-100 for 20 min, blocked with blocking solution at RT for 1 h, and incubated with primary antibodies overnight at 4°C. Sections were then immunostained by the ABC peroxidase method (Vector Laboratories) with diaminobenzidine (DAB) as the substrate and hematoxylin as the counterstain. For immunofluorescence staining, sections were incubated with primary antibodies overnight at 4°C after antigen retrieval with 0.01 M citrate buffer (pH 6.0) via microwave, incubated with Alexa Fluor 488 and 594 goat anti-mouse, anti-rabbit or anti-rat IgG (H+L) secondary antibodies (Invitrogen), and counterstained with DAPI to label nuclear DNA. Primary

antibodies included anti-MEX3A (Sigma, PRS4869, 1:400), E2F3 (Thermo Fisher, PA5-106407, 1:100), anti-Ki67 (Abcam, ab15580, 1:1000), anti-Mucin2 (Santa Cruz, sc-15334, 1:500), anti-ChgA (Thermo Fisher, PA5-18527, 1:100), anti-Olfm4 (Cell Signaling Technology, 39141, 1:800), anti-GFP (Abcam, ab13970, 1:800), anti-phospho-histone H2A.X (Cell Signaling Technology, 9718, 1:480 for immunohistochemistry, 1:400 for immunofluorescence), anti-cleaved Caspase3 (Cell Signaling Technology, 9664, 1:2000), anti-p21 (Santa Cruz, sc-817, 1:100), anti-p53 (Cell Signaling Technology, 2524, 1:1000), anti-non-phospho (Active) β-catenin (Ser45) (Cell Signaling Technology, 19807, 1:1000), anti-KLF4 (Abcam, ab214666, 1:1000; Abcam, ab215036, 1:2000), anti-c-Myc (Abcam, ab32072, 1:100), anti-CD44 (Cell Signaling Technology, 37259, 1:200; Proteintech, 15675-1-AP, 1:200), anti-E-cadherin (Proteintech, 20874-1-AP, 1:200), anti-Cytokeratin 20 (Abcam, ab109111, 1:75), anti-CDK2 (Abcam, ab32147, 1:50) and anti-Cyclin A2 (Abcam, ab181591, 1:500).

For BrdU staining, mice were intraperitoneally injected with BrdU solution at a concentration of 50 μg/g body weight. Deparaffinized sections were consecutively treated with a 1:1 mixture of 2 × SSC and formamide (Amresco, Solon, OH) at 65°C for 2 h, 2 × SSC at RT for 5 min, 1 M HCl at 4°C for 10 min, 2 M HCl at 37°C for 30 min, 0.1 M boric acid at RT for 10 min and washed in 1% PBST. Then sections were blocked with 5% normal goat serum/0.1 M glycine in 1% PBST at RT for 1 h and incubated with anti-BrdU antibody (Abcam, ab6326, 1:100) overnight at 4°C. The remaining steps were the same as for immunofluorescence staining. EdU staining was performed using the Click-iT EdU Alexa Flour 594 kit (Beyotime, C0078S) according to the manufacturer's instructions.

Confocal imaging

NCM460 cells were grown on a circular microscope cover glass (NEST, 801010), washed once with filtered PBS, and fixed in 4% PFA for 30 min at RT. Cells were blocked with 5% BSA in PBS at RT for 1 h after permeabilization with 1% Triton X-100 and then incubated with primary antibodies overnight at 4°C. The remaining steps were the same as those for immunofluorescence staining. Images were captured using a Leica laser scanning confocal microscope (Leica TCS SP8).

Flow cytometry and cell sorting

Intestinal crypt cells were isolated from fresh mouse intestine samples by incubation with 10 mM EDTA in PBS for 30 min at 4°C. The crypt fractions were collected by vigorous shaking, followed by filtration through a 70 μm cell strainer (BD Biosciences). The gathered crypt cells were centrifuged at 1200 rpm for 5 min and then digested with dispase (1 U/ml, STEMCELL Technologies). Single cell suspensions were passed through a 40 μm cell strainer (BD Biosciences) and stained with Fixable Viability Dye (eBioscience, 65-0863-14) for 20 min to remove dead cells. Flow cytometry analysis was performed on a BD FACS Aria 3.0. $Lgr5^{high}$ cells, $Lgr5^{low}$ cells and $Lgr5^{neg}$ cells were sorted by flow cytometry from *Lgr5-EGFP-IRES-Cre*^{ERT2} mice. $Lgr5^{high}$ cells were sorted by flow cytometry from *Mex3a*$^{+/+}$*;Lgr5-EGFP-IRES-Cre*^{ERT2} mice and *Mex3a*$^{-/-}$*;Lgr5-EGFP-IRES-Cre*^{ERT2} mice.

For cell cycle analysis, HCT116 cells transfected with corresponding plasmids were harvested, washed twice with cold PBS and then fixed at 4°C with 70% ethanol overnight. Fixed cells were washed twice with cold PBS and stained with PI solution (50 μg/ml, 0.2% Triton X-100, 100 μg/ml RNase A) with protection from light for 30 min at 4°C. Stained cells were analyzed by BD FACSVerse flow cytometry.

Organoid and single-cell culture

Isolation of intestinal crypts was performed as described above. Gathered crypts were washed twice with PBS and centrifuged at 700 rpm for 5 min. Supernatant was removed, and then crypts were resuspended in a 1:1 mixture of IntestiCult OGM (STEMCELL Technologies, 06005) and Matrigel (Corning, 356231) and plated into 48-well plates. After Matrigel polymerization, 200 µl OGM was added to each well. Medium was replaced every 2 days, and organoids were passaged every 4 days. For APKS organoid culture, medium was DMEM/F12 with 1 × B-27 (Gibco, 17504044), 1 × N-2 (Gibco, 17502048), 1 mM N-acetyl-cysteine (Sigma, A9165), 1% Pen Strep (Gibco, 15140122), 1 × GlutaMAX (Gibco, 35050061), and 10 mM HEPES (Gibco, 15630080). For propidium-iodide (PI) stained organoid cell-death assay, organoids were stained with 50 µg/ml PI.

For $Lgr5^{high}$ cell culture, a total of 20 000 sorted $Lgr5^{high}$ cells were collected into 1.5 ml tubes containing 2% FBS and 10 µM Y-27632 (STEMCELL Technologies) in DMEM/F12 medium. A total of 5000 cells per well were embedded in Matrigel and seeded in 48-well plates. After Matrigel polymerization, 200 µl OGM was added to each well. For the first three days, 10 µM Y-27632 was added to OGM medium, and medium was replaced every day.

Generation of APKS mouse tumor organoids

The colonic crypts from a $Kras^{LSL\text{-}G12D}$ mouse were extracted and used to establish the culture. $Kras^{G12D}$ mutation was then activated by transient transfection of *Salk-Cre* with pPGK-Puro (Addgene#11349) plasmids, followed with puromycin selection for 3 days. The *APC*, *P53*, *Smad4* mutations were introduced by CRISPR/Cas9 editing. Specifically, sgRNAs of *APC*, *P53* and *Smad4* were cloned into PX330 plasmid (Addgene#42230) and transiently transfected into the

puromycin selected tumoroids. One week after the transient transfection, the tumoroids with *APC*, *P53* and *Smad4* mutations were selected by removing R-spondin, adding Nutlin-3 and removing Noggin from the culture media, respectively. Ten subclones were picked from the engineered bulk tumoroids, conditional PCR and Sanger sequencing were used to verify the mutations in each subclone. Subclones with recombined *LSL-Kras*^{G12D} allele, and verified *APC*, *P53* and *Smad4* mutations were used for downstream experiments. The sequences of sgRNAs used for CRISPR/Cas9 editing and sequences of mutated genes are listed in **Figure S9B-D**.

Chromatin immunoprecipitation (ChIP) assay

ChIP assay was performed using the SimpleChIP enzymatic chromatin immunoprecipitation kit (Cell Signaling Technology, 9002) according to the manufacturer's instructions. Harvested CT26 cells were crosslinked with 1% (*v/v*) formaldehyde for 10 min. After nuclei preparation, micrococcal nuclease was used to digest DNA to a length of 150-900 bp. The immunoprecipitation preparations were divided for input control and were incubated with anti-E2f3, anti-Histone H3 (as a positive control) and anti-IgG (as a negative control) at 4°C overnight. The obtained genomic DNA was quantified by qRT-PCR with primers specific for E2f3 binding elements of *Mex3a* promoter regions.

qRT-PCR analysis

Total RNA was extracted from sorted cells, cell lines, organoids and mouse intestinal tissues using TRIzol reagent (Life Technologies) according to the manufacturer's instructions. To detect mRNA levels, reverse transcription was carried out using oligo (dT) primers. qRT-PCR was performed using LightCycler 480 SYBR Green I Master Mix on a LightCycler 480 Real-Time PCR System

(Roche, Mannheim, Germany). Relative expression was calculated based on the $2^{-\Delta\Delta Ct}$ method, and *Gapdh* was used as the internal control. Primers for qRT-PCR analysis are included in **Table S2**.

RNA-Seq analysis

Intestinal crypt cells were isolated from the intestines of four KO mice and four littermate controls by incubation with 10 mM EDTA, 10 mM HEPES and 2% FBS in HBSS for 15 min at 37°C. Crypt fractions were collected by vigorous shaking and filtered through a 70 μm cell strainer. Gathered crypt cells were centrifuged at 1200 rpm for 5 min. Total RNA was isolated from collected crypt cells using TRIzol reagent according to the manufacturer's instructions. RNA samples were sent to Novogene Co., Ltd. for library preparation and sequencing on the Illumina NovaSeq 6000 platform. The data were analyzed online on the NovoMagic data analysis cloud platform (www.magic.novogene.com) or using R software. RNA-Seq data has been submitted to the GEO repository under accession number GSE179493.

Cell culture and transfections

HCT116 and HEK293FT cell lines were purchased from the American Type Culture Collection (ATCC) (Manassas, VA) and cultured in IMDM and DMEM supplemented with 10% FBS, respectively. NCM460 cell line was purchased from the Innovative Life Science Solutions (INCELL) (San Antonio, TX). Caco-2 cell line was purchased from ATCC and cultured in DMEM supplemented with 20% FBS. CT26 cell line was purchased from ATCC and cultured in RPMI 1640 supplemented with 10% FBS. All cell lines were tested and confirmed to be free of mycoplasma infection. For Caco-2 3D culture, 48-well plates were coated with 70 ul/well of

matrigel. After Matrigel polymerization, 6×10^3 cells/well suspension plus 2% matrigel was seeded on top. Medium was replaced every 2 days. For tumor sphere formation, HCT116 cells were cultured in serum-free DMEM/F-12 medium, containing 2% B27 (Gibco, 17504044), 20 ng/mL EGF (R&D, 236-EG) and 20 ng/mL bFGF (R&D, 233-FB) in 6-well ultra-low attachment culture plates.

Transient transfections were performed using Lipofectamine 2000 reagent (Invitrogen, 11668019) with 2 μg vector or negative control vector in one well of a 6-well plate according to the manufacturer's protocol. For HCT116 and NCM460 cells, media were changed at 4 h posttransfection.

Clonogenic assay

Following radiation, HCT116 cells were re-plated at a cell density of 1200 per well in 6-well plates. After 8 days of incubation, cells were fixed with 4% PFA and stained with 0.4% crystal violet. Then, numbers of colonies were counted.

Plasmid construction

Full-length human MEX3A, E2F3, KLF4 and mouse E2f3 constructs were cloned into a pcDNA3.1 vector. shMEX3A and shKLF4 were subcloned into pGPU6-GFP vector (**Table S3**). For luciferase assays, construct including the 247 bp or 273 bp 3'-UTR sequence of *KLF4* was cloned into psiCHECK-2 vector, and construct including 2 kb *Mex3a* promoter sequence was cloned into pGL3-Basic vector. All mutants were generated through site-directed mutagenesis (BGI, Shenzhen, China). All constructs were verified by performing DNA sequencing.

Luciferase assays

The sequence for *Mex3a* is located on chromosome 3 (NC_000069.7, base pairs 88439253…88448701) in the mouse genome. In luciferase assay for *Mex3a* promoter activity performed in this study, *Mex3a* promoter was identified as an approximately 2 kb region upstream of the transcript start site (TSS), which is located at chromosome 3 (NC_000069.7, base pairs 88437253…88439252); this sequence was cloned into the pGL3-Basic reporter constructs. Binding sites 1 and 2 of E2f3 are located at base pairs 88439153-88439167 and 88438895-88438909, respectively. The firefly and Renilla luciferase activities were measured after 24 h of transfection using Dual-Glo luciferase assay kit (Promega) according to the manufacturer's instructions.

For dual-luciferase activity assay, *KLF4* 3'-UTR fragment containing binding site 5'-TGAGTCTTGGTTCTA-3' or 5'-TGAGAATTAAGTTTTA-3' was cloned into psiCHECK-2 reporter constructs. After 24 h of transfection, firefly and Renilla luciferase activities were measured with a Dual-Glo luciferase assay kit (Promega, E2920) according to manufacturer's instructions.

Anchorage-independent growth

A 60 mm cell culture dish was coated with 3 ml of a 1:1 mixture of 1.2% agarose and IMDM supplemented with 20% FBS. After mixture solidification, 1×10^4 transfected HCT116 cells per dish were collected and resuspended in 1 ml of a 1:1 mixture of 0.7% agarose and IMDM supplemented with 20% FBS and then transferred to coated 60 mm cell culture dish. After mixture solidification, 1 ml IMDM supplemented with 10% FBS was added to the surface of coated 60 mm cell culture dish and replaced every 3 days for a total of 3 weeks of culture.

Cell proliferation assay

For this assay, 3000 cells per well were seeded in 96-well plate. After 24 h of transfection, Enhanced Cell Counting Kit-8 (Beyotime, C0042) was used to detect cell proliferation according to manufacturer's instructions. For quantification, 10 µl of reagent was added to each individual well and mixed at 37°C for 1 h. Absorbance was measured using Spark Multimode Microplate Reader (Tecan, Switzerland).

RNA stability assay

HCT116 cells transfected with pcDNA3.1 empty vector or pcDNA3.1-MEX3A plasmids were exposed to 5 µg/ml of Actinomycin D (MedChemExpress, HY-17559). RNA was measured at 0, 2, 4, 6 and 8 hours using qRT-PCR.

Nuclear and cytoplasmic protein extraction

Transfected NCM460 cells were washed once with cold PBS, collected in 500 µl PBS per well by scraping plate surface, and centrifuged at 1000 rpm for 5 min. Nuclear and cytoplasmic protein were isolated from harvested cells using Nuclear and Cytoplasmic Protein Extraction Kit (Beyotime, P0027) according to manufacturer's instructions. Protein concentration was determined with a BCA Kit (Beyotime, P0011). Histone H3 was used as an internal control for nuclear fraction, and GAPDH was used as an internal control for cytoplasmic fraction.

Western blotting

Western blotting assays were performed according to standard procedures. Fresh tissues were homogenized using RIPA buffer (Beyotime, P0013C) in the presence of protease and phosphatase inhibitor cocktails (Roche), followed by treatment with a homogenizer (T10 basic, IKA). Proteins were measured by BCA protein assay kit (Beyotime) and denatured. Total protein samples (30 μg) were separated on 8-12% SDS-PAGE gels and transferred to PVDF membranes (GE Healthcare). Then, PVDF membranes were blocked with 5% nonfat dry milk at RT for 1 h and incubated with primary antibodies overnight at 4°C. Images were taken using a chemiluminescence imaging system (SageCreation, Beijing). Relative protein band intensity was quantified by ImageJ software (U.S. National Institutes of Health, Bethesda, MD, USA). The following antibodies were used: anti-β-Actin (YEASEN, 30101, 1:5000), anti-α-Tubulin (Beyotime, AF0001, 1:5000), anti-MEX3A (Sigma, PRS4869, 1:1000), anti-E2F3 (Santa Cruz, sc-28308, 1:500), anti-Axin2 (Abcam, ab109307, 1:1000), anti-LBH (Santa Cruz, sc-161791, 1:100), anti-Tcf-1 (Santa Cruz, sc-271453, 1:500), anti-Cyclin D1 (Cell Signaling Technology, 2978, 1:1000), anti-c-Myc (Abcam, ab32072, 1:1000), anti-KLF4 (Abcam, ab214666, 1:1000; Abcam, ab215036, 1:1000), anti-Histone H3 (Cell Signaling Technology, 4499, 1:2000), and anti-GAPDH (Beyotime, AF0006, 1:5000).

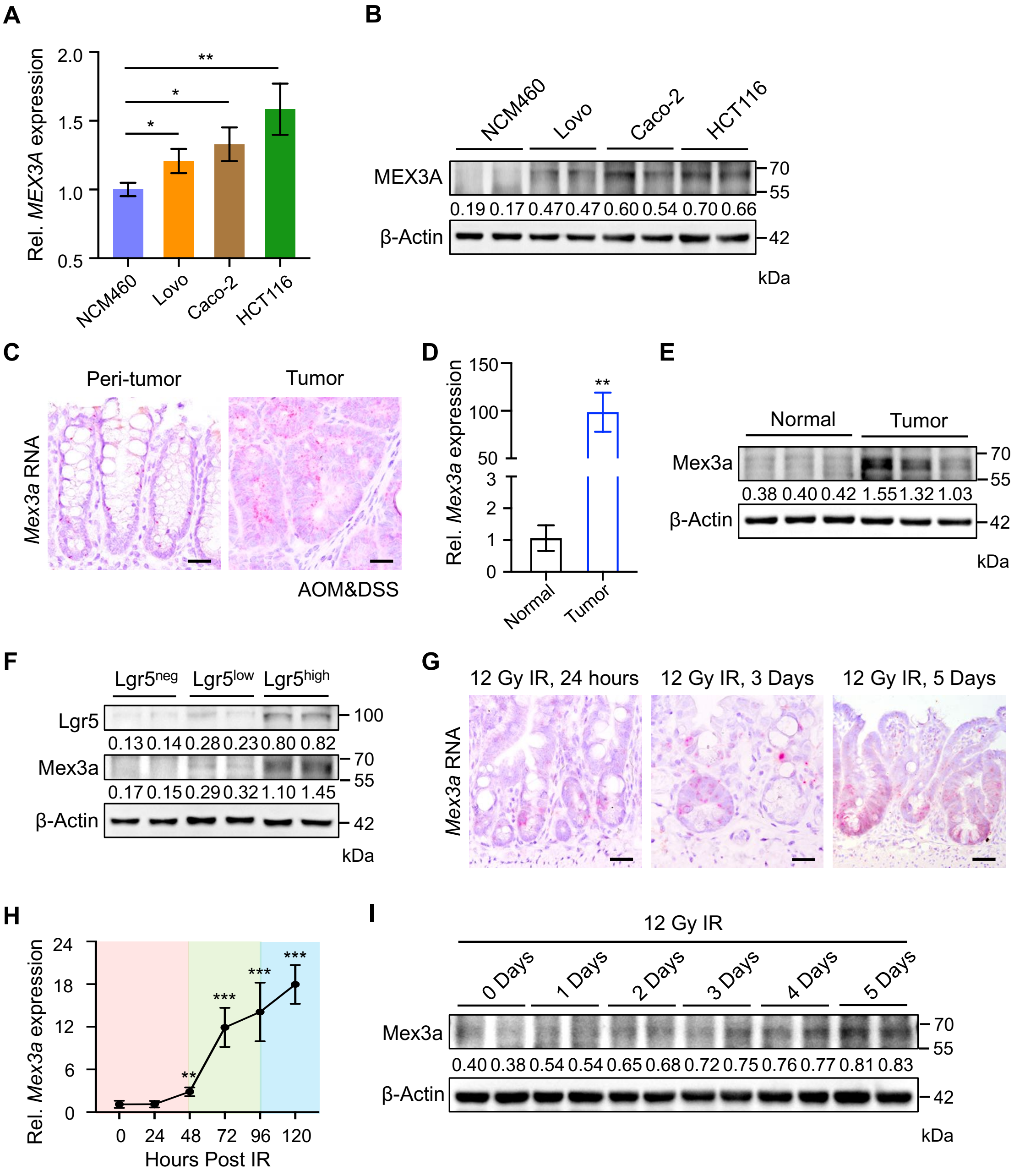

Figure S1. ***Mex3a*** **is upregulated in CRC and regenerative foci. A-B,** qRT-PCR (**A**, n = 3) and Western blotting (**B**) analysis for MEX3A in normal colorectal epithelial cell and different human colon cancer cell lines. β-Actin was used as a loading control. **C,** *In situ* hybridization for *Mex3a* with RNAscope probe in mouse colon peritumor and tumor tissues from AOM-DSS model. Scale bar: 25 μm. **D-E,** qRT-PCR (**D**, n = 3) and Western blotting (**E**) analysis of Mex3a in normal mouse colon tissues and colon tumors from AOM-DSS model. β-Actin was used as a loading control. **F,** Western blotting for Mex3a and Lgr5 in sorted $Lgr5^{neg}$, $Lgr5^{low}$ and $Lgr5^{high}$ cells. β-Actin was used as a loading control. **G,** *In situ* hybridization for *Mex3a* with RNAscope probe in mouse intestinal crypts 24 hours, 3 days or 5 days after 12 Gy γ-radiation. Scale bar: 25 μm. **H-I,** qRT-PCR (**H**, n = 3 biological replicates at each time point) and Western blotting (**I**) analysis

showing dynamic changes of *Mex3a* after exposure to 12 Gy γ-radiation. Different background colors indicate different phases of regenerative response. Red: DNA damage phase. Green: proliferative phase. Blue: normalization phase. β-Actin was used as a loading control. Data are presented as the mean ± SD. *$P < 0.05$; **$P < 0.01$; ***$P < 0.001$.

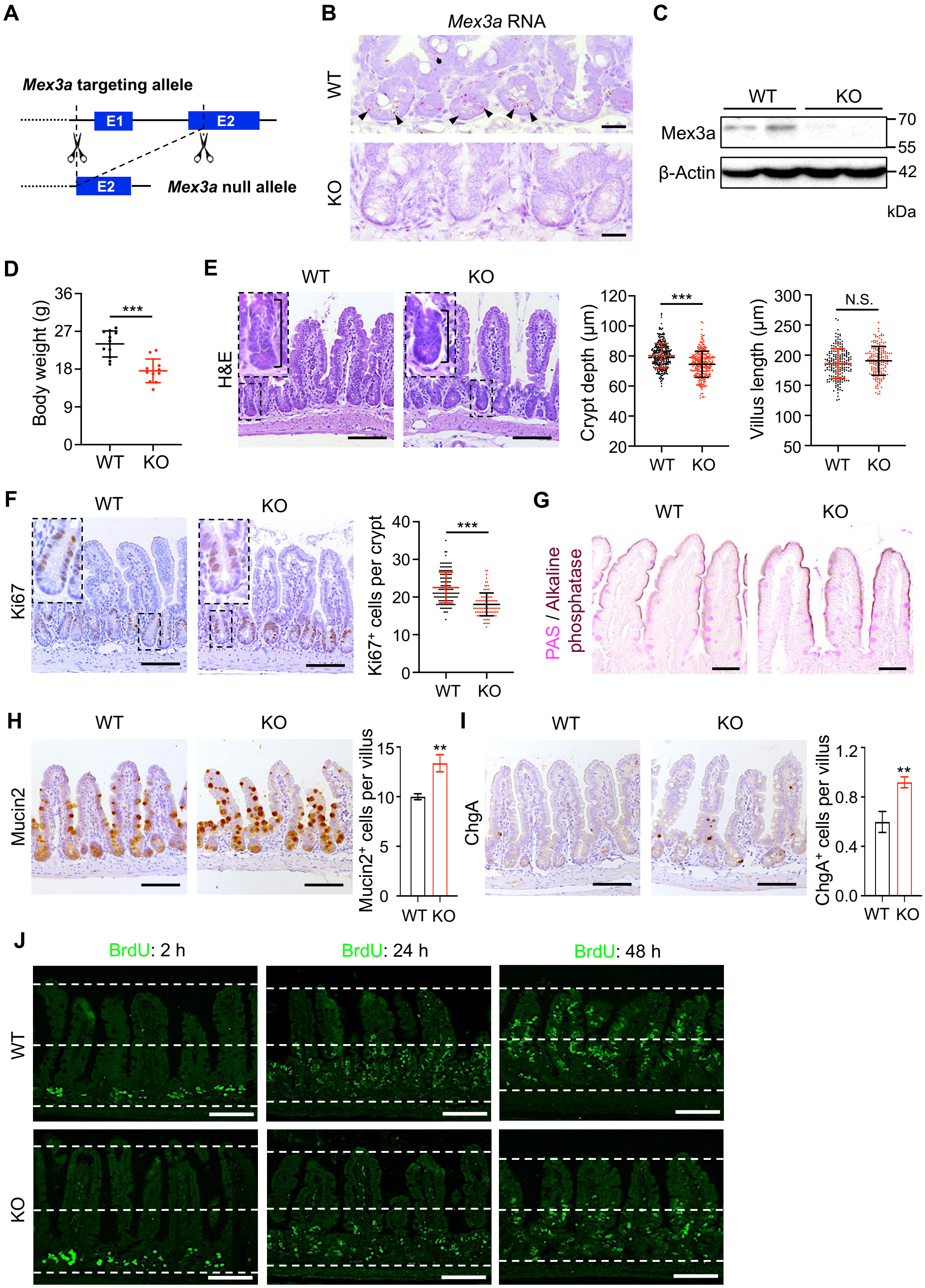

Figure S2. Deletion of *Mex3a* leads to disrupted intestinal homeostasis. A, Schematics of *Mex3a* genomic locus and strategy for generating *Mex3a* knockout (KO) mice. **B,** *In situ*

hybridization for *Mex3a* with RNAscope probe in intestines from wild-type (WT) and KO mice, showing that *Mex3a* was completely deleted. n = 3. Scale bar: 25 μm. **C,** Western blotting for Mex3a in intestinal tissues from WT and KO mice. β-Actin was used as a loading control. **D,** Body weight of 8-week-old WT and KO mice. n = 11. **E,** Histology of intestines from WT and KO mice. Crypt depth and villus length were quantified. WT, n = 304 crypts, n = 197 villi, n = 5 mice; KO, n = 261 crypts, n = 173 villi, n = 5 mice. Scale bar: 100 μm. **F,** Immunohistochemistry for Ki67 and quantification of $Ki67^+$ cells per crypt in ileum from WT and KO mice. WT, n = 138 crypts, 3 mice; KO, n = 107 crypts, 3 mice. Scale bar: 100 μm. **G,** Representative images of PAS-alkaline phosphatase staining in ileum from WT and KO mice. n = 3. Scale bar: 50 μm. **H**-**I,** Immunohistochemistry for Mucin2 (**H**) and ChgA (**I**) in ileum from WT and KO mice. $Mucin2^+$ cells and $ChgA^+$ cells per crypt-villus architecture were quantified. n = 3. Scale bar: 100 μm. **J,** Immunofluorescence for BrdU in ileum from WT and KO mice at indicated timepoints after one does of BrdU pulse. The dashed lines indicate top of the villi, middle line of the intestine, and base of the crypt. Scale bar: 100 μm. Data are presented as the mean ± SD. *$P < 0.05$; **$P < 0.01$; ***$P < 0.001$.

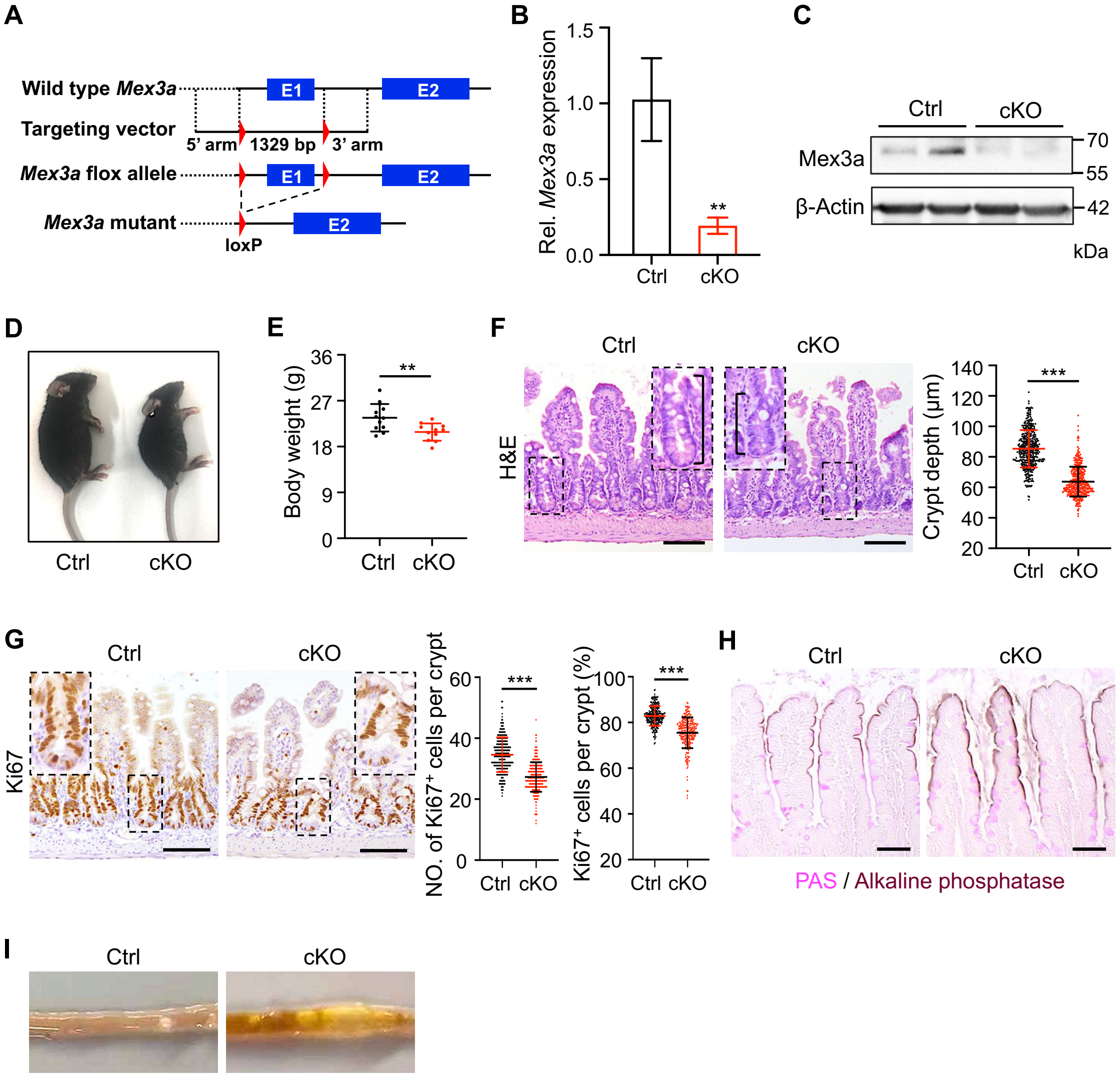

Figure S3. Deletion of *Mex3a* within intestinal epithelium phenocopies those of *Mex3a* constitutive KO mice. A, Schematic for generating *Mex3a* floxed alleles. **B,** qRT-PCR for *Mex3a* in intestinal tissues from *Villin-Cre;*$Mex3a^{fl/fl}$ (cKO) and littermate control (Ctrl) mice. n = 3. **C,** Western blotting for Mex3a in intestinal tissues from Ctrl and cKO mice. β-Actin was used as a loading control. **D,** Gross images of Ctrl and cKO mice at age of 8 weeks. **E,** Body weights of Ctrl and cKO mice at age of 8 weeks. n = 11. **F,** Histological images and quantification of crypt depth in intestinal tissues from Ctrl and cKO mice. Ctrl, n = 404 crypts, 5 mice; cKO, n = 369 crypts, 5 mice. Scale bar: 100 μm. **G,** Immunohistochemistry for Ki67 in ileum tissues from Ctrl and cKO mice. The quantities and proportions of Ki67$^+$ cells per crypt were determined. Ctrl, n = 277 crypts, 3 mice; cKO, n = 281 crypts, 3 mice. Scale bar: 50 μm. **H,** Representative PAS-alkaline phosphatase staining in Ctrl and cKO mouse ileum tissues. n = 3. Scale bar: 50 μm. **I,** Representative macroscopic images of intestines from Ctrl and cKO mice. n = 4. Data are presented as the mean ± SD. *$P < 0.05$; **$P < 0.01$; ***$P < 0.001$.

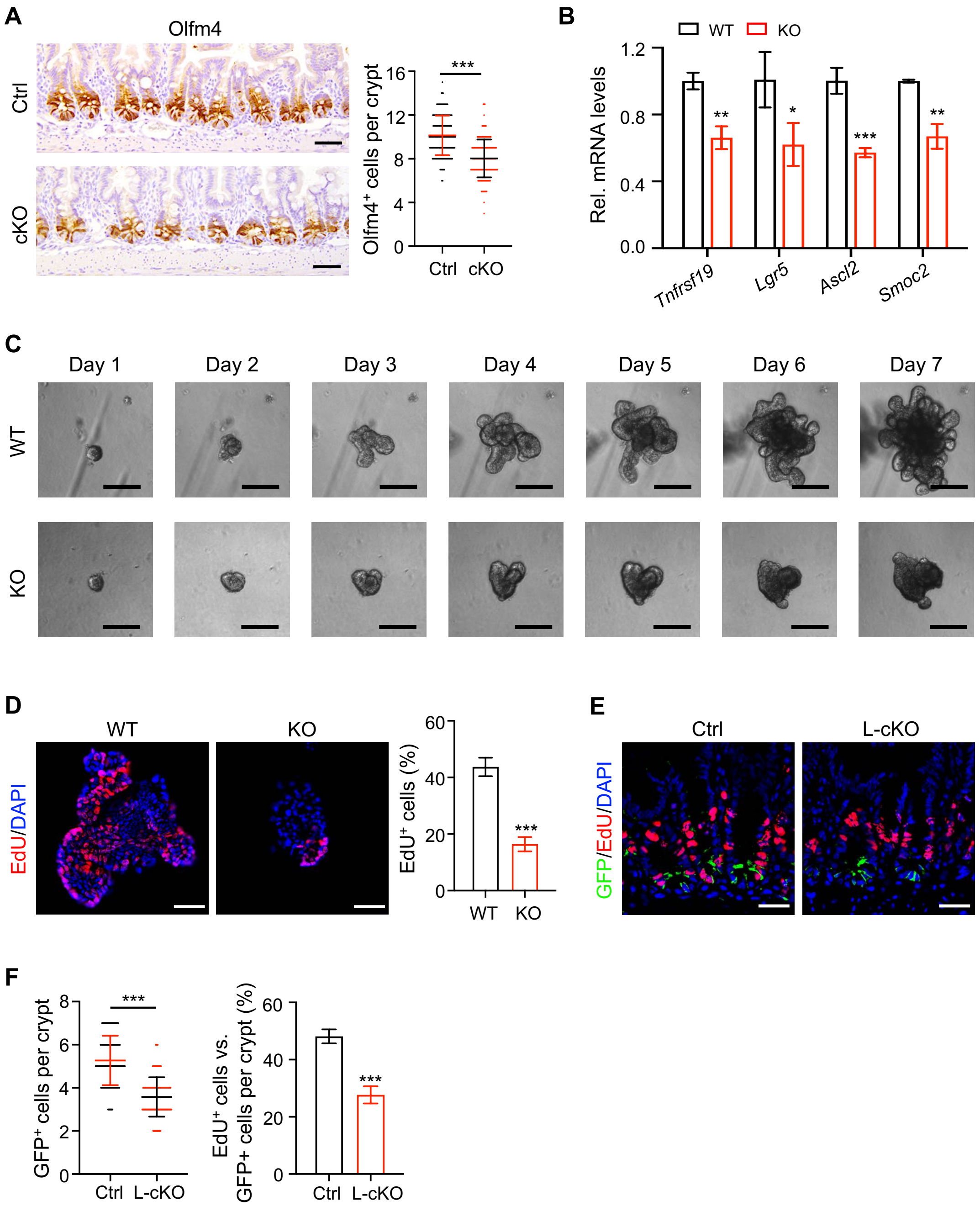

Figure S4. Deletion of *Mex3a* leads to a reduction in the number of ISCs. A, Immunochemistry for Olfm4 in intestinal crypts from control (Ctrl) and cKO mice. Number of Olfm4$^+$ cells per crypt was quantified. Ctrl, n = 162 crypts, 3 mice; cKO, n = 157 crypts, 3 mice. Scale bar: 50 μm. **B,** qRT-PCR for crypt base columnar cell (CBC) marker genes *Tnfrsf19*, *Lgr5*, *Ascl2* and *Smoc2* in intestinal crypts from wild-type (WT) and KO mice. n = 3. **C,** The successive images of crypts purified from WT and KO mouse organoid cultures at indicated timepoints. Scale bar: 200 μm. **D,** Immunofluorescence for EdU in intestinal organoids cultured 3 days after seeding. Percentage of EdU$^+$ cells was quantified. n = 3. Scale bar: 50 μm. **E,** Double immunofluorescence for GFP and EdU in ileum from $Lgr5^{EGFP\text{-}CreERT2}$;$Mex3a^{fl/fl}$ (L-cKO) and littermate control (Ctrl) mice. Scale

bar: 25 μm. **F,** Number of GFP^{+} cells per crypt and percentage of EdU^{+}GFP^{+} cells versus GFP^{+} cells per crypt in panel E were quantified. Control, n = 408 crypts, 3 mice; L-cKO, n = 328 crypts, 3 mice. Data are presented as the mean ± SD. *$P < 0.05$; **$P < 0.01$; ***$P < 0.001$.

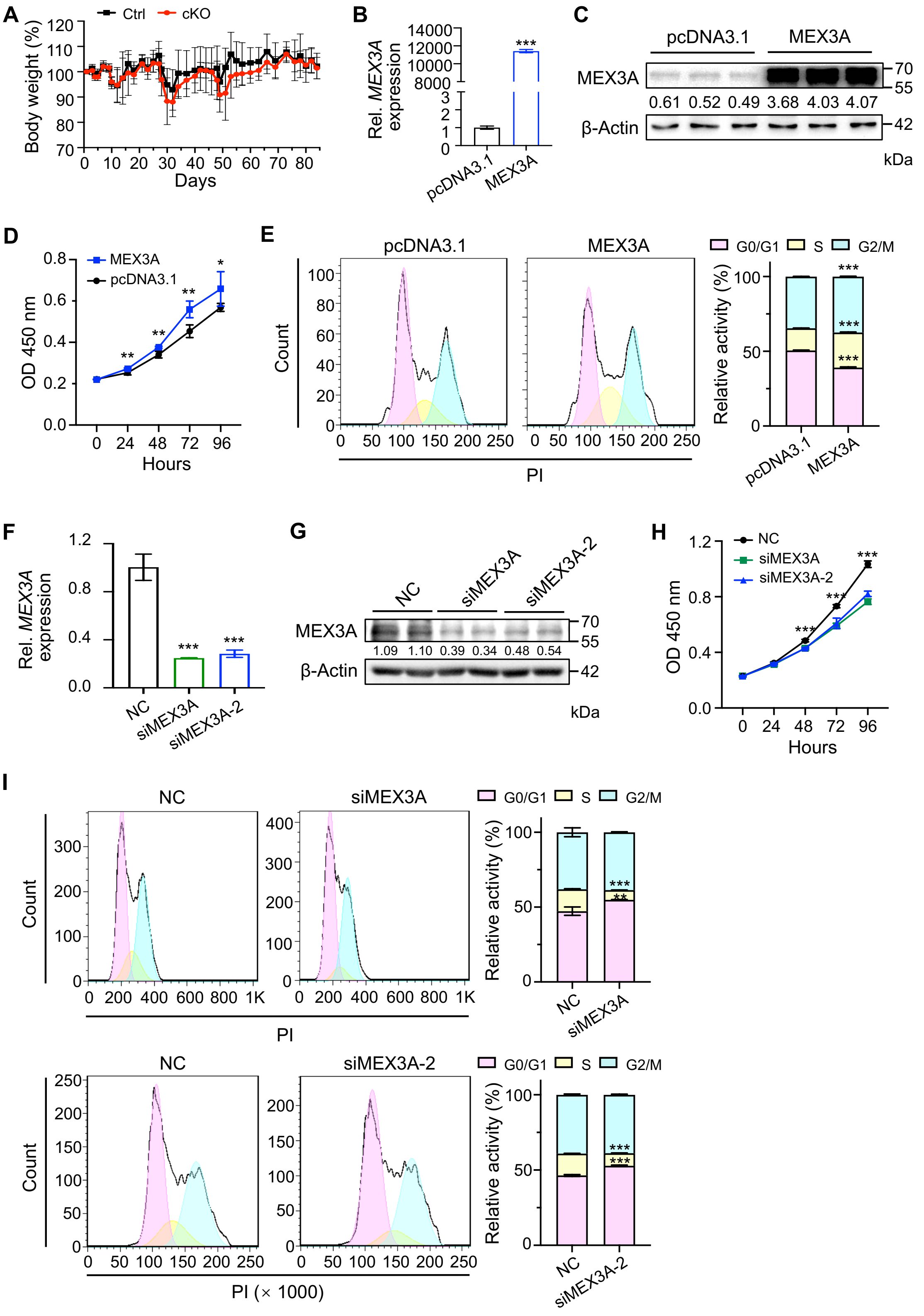

Figure S5. MEX3A promotes the growth of colon cancer cells *in vitro*. A, Body weight changes of control (Ctrl) and cKO mice during AOM-DSS-induced tumor development. **B-C,** qRT-PCR

analysis (**B**) and Western blotting (**C**) of MEX3A in HCT116 cells transfected with pcDNA3.1-MEX3A plasmids. n = 3. β-Actin was used as a loading control. **D,** Growth curve of HCT116 cells transfected with pcDNA3.1-MEX3A plasmids over time. n = 5. **E,** Cell cycle analysis with flow cytometry for HCT116 cells 24 hours after transfection with pcDNA3.1-MEX3A plasmids. n = 3. **F-G,** qRT-PCR analysis (**F**) and Western blotting (**G**) of MEX3A in HCT116 cells 24 hours after *MEX3A* siRNAs treatment. The sequences of *MEX3A* siRNAs are shown in Supplementary Table S4. β-Actin was used as a loading control. **H,** Growth curve of HCT116 cells over time after *MEX3A* siRNAs transfection. n = 5. **I,** Flow cytometry assay for the cell cycle pattern of HCT116 cells treated with *MEX3A* siRNAs. n = 3. Data are presented as the mean ± SD. *$P < 0.05$; **$P < 0.01$; ***$P < 0.001$.

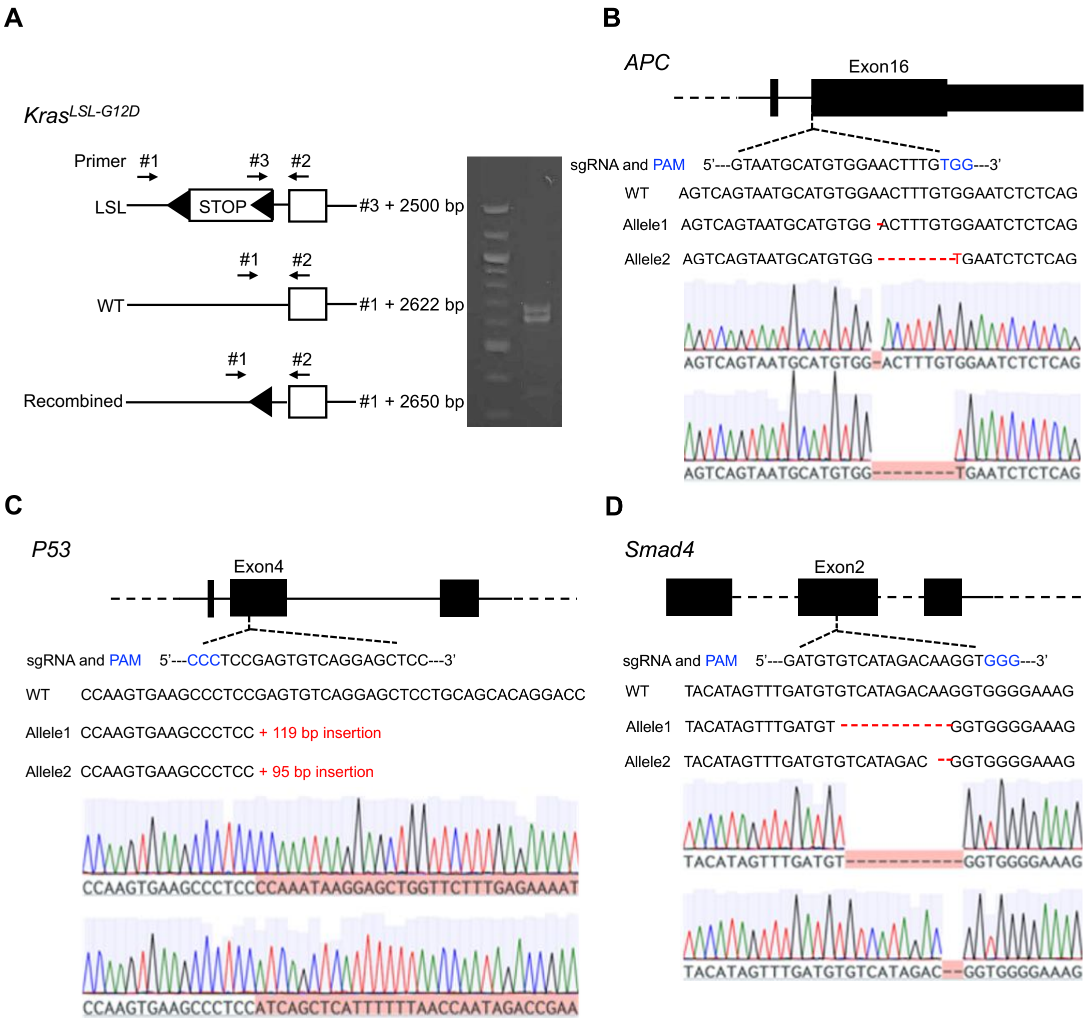

Figure S6. Generation of APKS mouse tumor organoids. A, Strategy for *Kras*^{G12D} mutation and genotyping of *Kras*$^{LSL\text{-}G12D}$ by PCR. **B-D,** *APC* mutations (**B**), *P53* mutations (**C**) and *Smad4* mutations (**D**) generated by CRISP/Cas9 system.

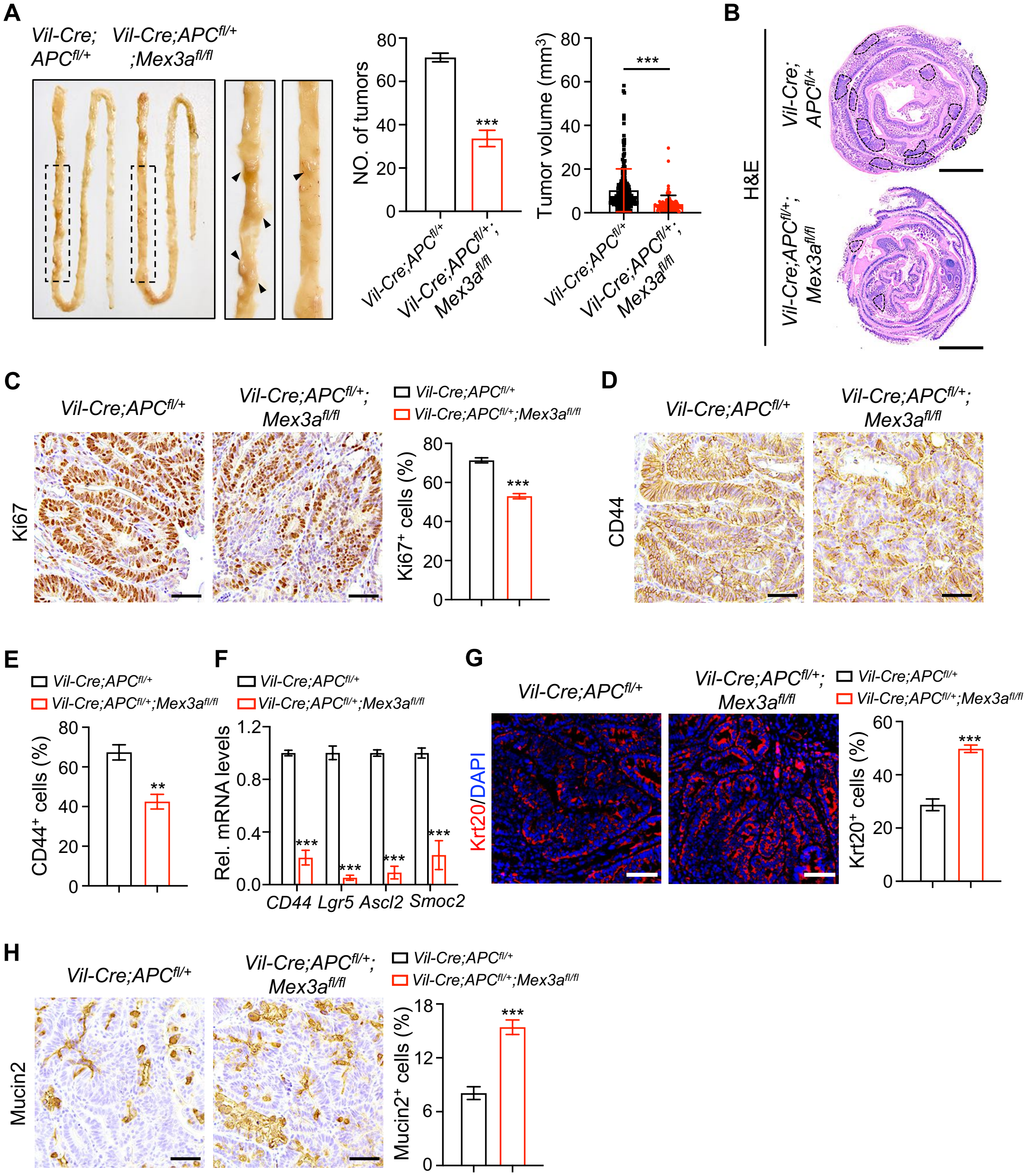

Figure S7. Deletion of *Mex3a* reduces tumor growth in *Vil-Cre;APC*$^{fl/+}$ mice. A, Gross images of intestine resected from *Vil-Cre;APC*$^{fl/+}$ and *Vil-Cre;APC*$^{fl/+}$*;Mex3a*$^{fl/fl}$ mice at 4 months of age. Arrowheads point to tumors. Number of tumors per mouse and tumor volume were quantified. *Vil-Cre;APC*$^{fl/+}$: n = 213 tumors from 3 mice. *Vil-Cre;APC*$^{fl/+}$*;Mex3a*$^{fl/fl}$: n = 101 tumors from 3 mice. **B,** Representative histological images of small intestine from 4-month-old *Vil-Cre;APC*$^{fl/+}$ and *Vil-Cre;APC*$^{fl/+}$*;Mex3a*$^{fl/fl}$ mice. n = 3. Scale bar: 2 mm. **C,** Immunohistochemistry for Ki67 in intestinal tumors from *Vil-Cre;APC*$^{fl/+}$ and *Vil-Cre;APC*$^{fl/+}$*;Mex3a*$^{fl/fl}$ mice. Percentage of Ki67^{+} cells was quantified. n = 3. Scale bar: 50 µm. **D-E,** Representative immunohistochemical images for CD44 in intestinal tumors from *Vil-Cre;APC*$^{fl/+}$ and *Vil-Cre;APC*$^{fl/+}$*;Mex3a*$^{fl/fl}$ mice (**D**).

Percentage of CD44^{+} cells was quantified (**E**). n = 3. Scale bar: 50 µm. **F,** qRT-PCR for cancer stem cell marker genes *CD44*, *Lgr5*, *Ascl2* and *Smoc2* in intestinal tumors from *Vil-Cre;APC*$^{fl/+}$ and *Vil-Cre;APC*$^{fl/+}$*;Mex3a*$^{fl/fl}$ mice. n = 3. **G-H,** Immunofluorescence for Krt20 (**G**) and immunohistochemistry for Mucin2 (**H**) in intestinal tumors from *Vil-Cre;APC*$^{fl/+}$ and *Vil-Cre;APC*$^{fl/+}$*;Mex3a*$^{fl/fl}$ mice. Percentage of Krt20^{+} cells and Mucin2^{+} cells were quantified. n = 3. Scale bar: 50 µm. Data are presented as the mean ± SD. **P* < 0.05; ***P* < 0.01; ****P* < 0.001.

339

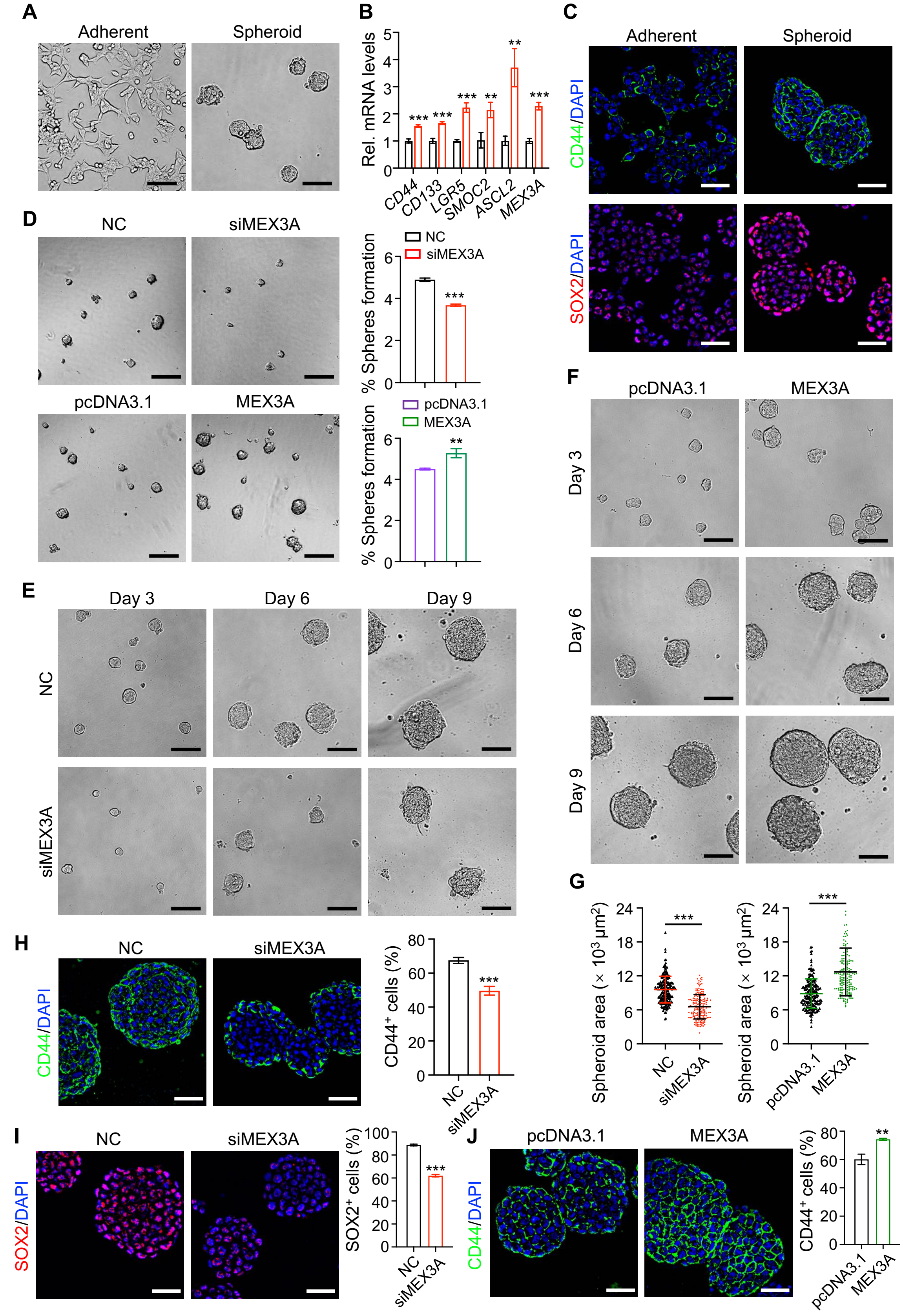

A
Adherent
Spheroid
B
Rel. mRNA levels
CD44
CD133
LGR5
SMOC2
ASCL2
MEX3A
C
Adherent
Spheroid
CD44/DAPI
SOX2/DAPI
D
NC
siMEX3A
pcDNA3.1
MEX3A
% Spheres formation
E
Day 3
Day 6
Day 9
NC
siMEX3A
F
pcDNA3.1
MEX3A
Day 3
Day 6
Day 9
G
Spheroid area (× 10³ μm²)
NC
siMEX3A
pcDNA3.1
MEX3A
H
NC
siMEX3A
CD44/DAPI
CD44⁺ cells (%)
I
NC
siMEX3A
SOX2/DAPI
SOX2⁺ cells (%)
J
pcDNA3.1
MEX3A
CD44/DAPI
CD44⁺ cells (%)

Figure S8. MEX3A is critical for colony formation and growth of CSCs. A, Representative gross images of HCT116 cells cultured in adherent (Left) and serum-free environment (Right). Scale bar: 100 μm. **B,** qRT-PCR for CSC marker genes in adherent cells and colorectal cancer stem-like cells. n = 3. **C,** Immunofluorescence for CSC marker genes CD44 and SOX2 in adherent cells and colorectal cancer stem-like cells. Scale bar: 25 μm. **D,** Representative gross images of tumor spheroids formed by HCT116 cells upon *MEX3A* knockdown or overexpression. The HCT116 cells were transfected with *MEX3A* siRNA or pcDNA3.1-MEX3A plasmids. 24 hours after transfection, the cells were seeded into ultra-low attachment culture plates with serum-free medium for 4 days. The percentage of growing tumor spheroids were quantified. n = 3. Scale bar: 200 μm. **E-F,** Growth of tumor spheroids over time formed by HCT116 cells after transfection with *MEX3A* siRNA (**E**) or pcDNA3.1-MEX3A plasmids (**F**). n = 3. Scale bar: 200 μm. **G,** Quantification of the spheroid area in panel E and F. **H-I,** Immunofluorescence for CSC markers CD44 (**H**) and SOX2 (**I**) in tumor spheroids formed by HCT116 cells after transfection with *MEX3A* siRNA. Percentage of $CD44^+$ cells and $SOX2^+$ cells were quantified. n = 3. Scale bar: 25 μm. **J,** Immunofluorescence for CD44 in tumor spheroids formed by HCT116 cells after transfection with pcDNA3.1-MEX3A plasmids. Percentage of $CD44^+$ cells was quantified. n = 3. Scale bar: 25 μm. Data are presented as the mean ± SD. *$P < 0.05$; **$P < 0.01$; ***$P < 0.001$.

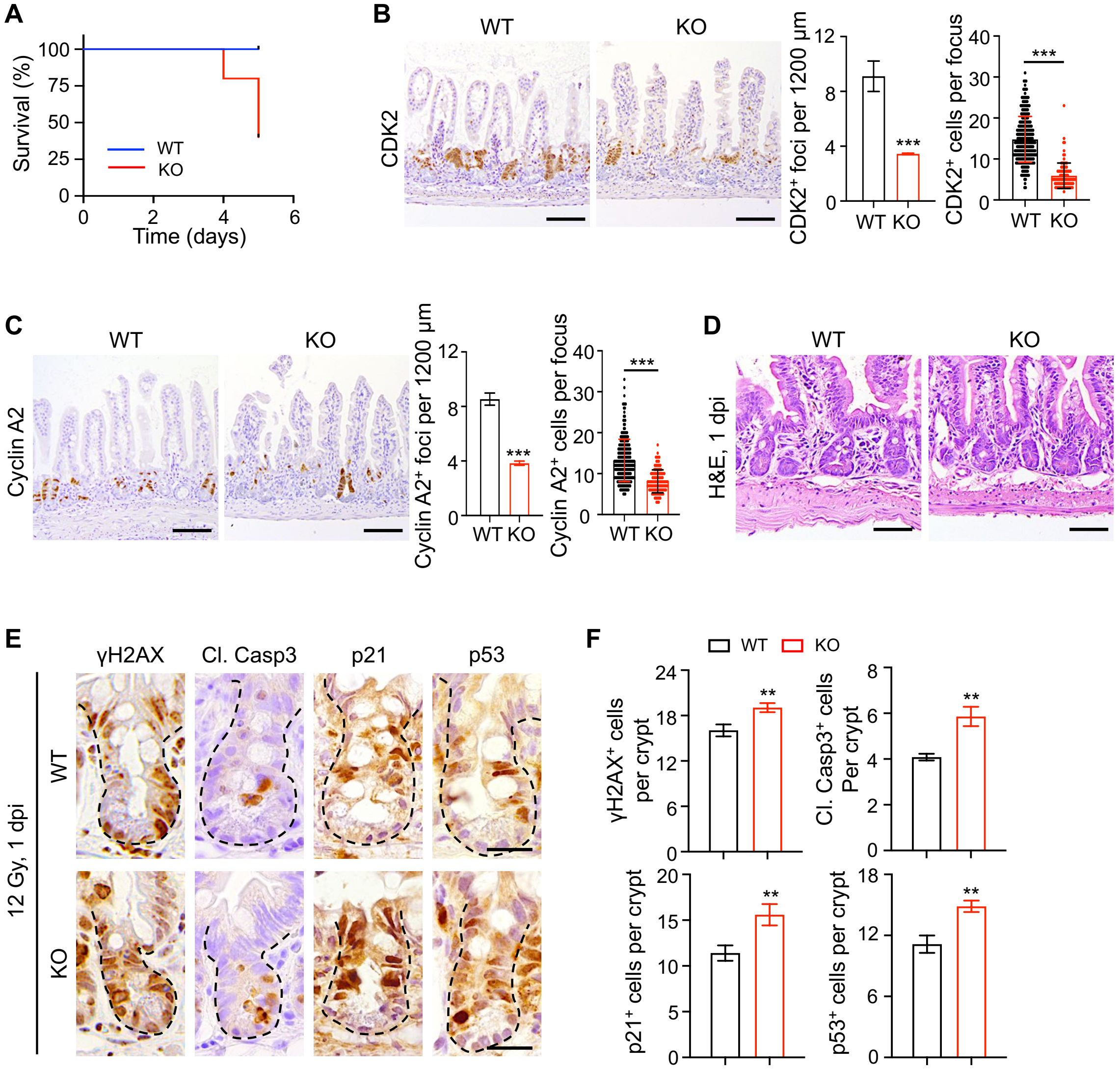

Figure S9. Deletion of Mex3a sensitizes ISCs to irradiation. A, Kaplan-Meier curve of 12 Gy-irradiated wild-type (WT) and KO mice. n = 5. **B,** Immunohistochemistry for CDK2 in ileum from wild-type (WT) and KO mice 3 days postirradiation. $CDK2^+$ regenerative foci per 1200 μm and number of $CDK2^+$ cells per regenerative focus were quantified. n = 3. Scale bar: 100 μm. **C,** Representative immunohistochemical images of Cyclin A2 in ileum from WT and KO mice 3 days postirradiation. Cyclin $A2^+$ regenerative foci per 1200 μm and number of Cyclin $A2^+$ cells per regenerative focus were quantified. n = 3. Scale bar: 100 μm. **D,** Histological images of ileum tissues from WT and KO mice 24 hours after 12 Gy γ-radiation. n = 3. Scale bar: 50 μm. **E,** Representative immunohistochemical images of γH2AX, cleaved Caspase3, p21 and p53 in ileum tissues from WT and KO mice 24 hours after 12 Gy γ-radiation. Scale bar: 25 μm. **F,** Quantification of $\gamma H2AX^+$ cells, cleaved $Casp3^+$ cells, $p21^+$ cells and $p53^+$ cells per crypt in panel E. n = 3. Data are presented as the mean ± SD. *$P < 0.05$; **$P < 0.01$; ***$P < 0.001$ (Student's t-test).

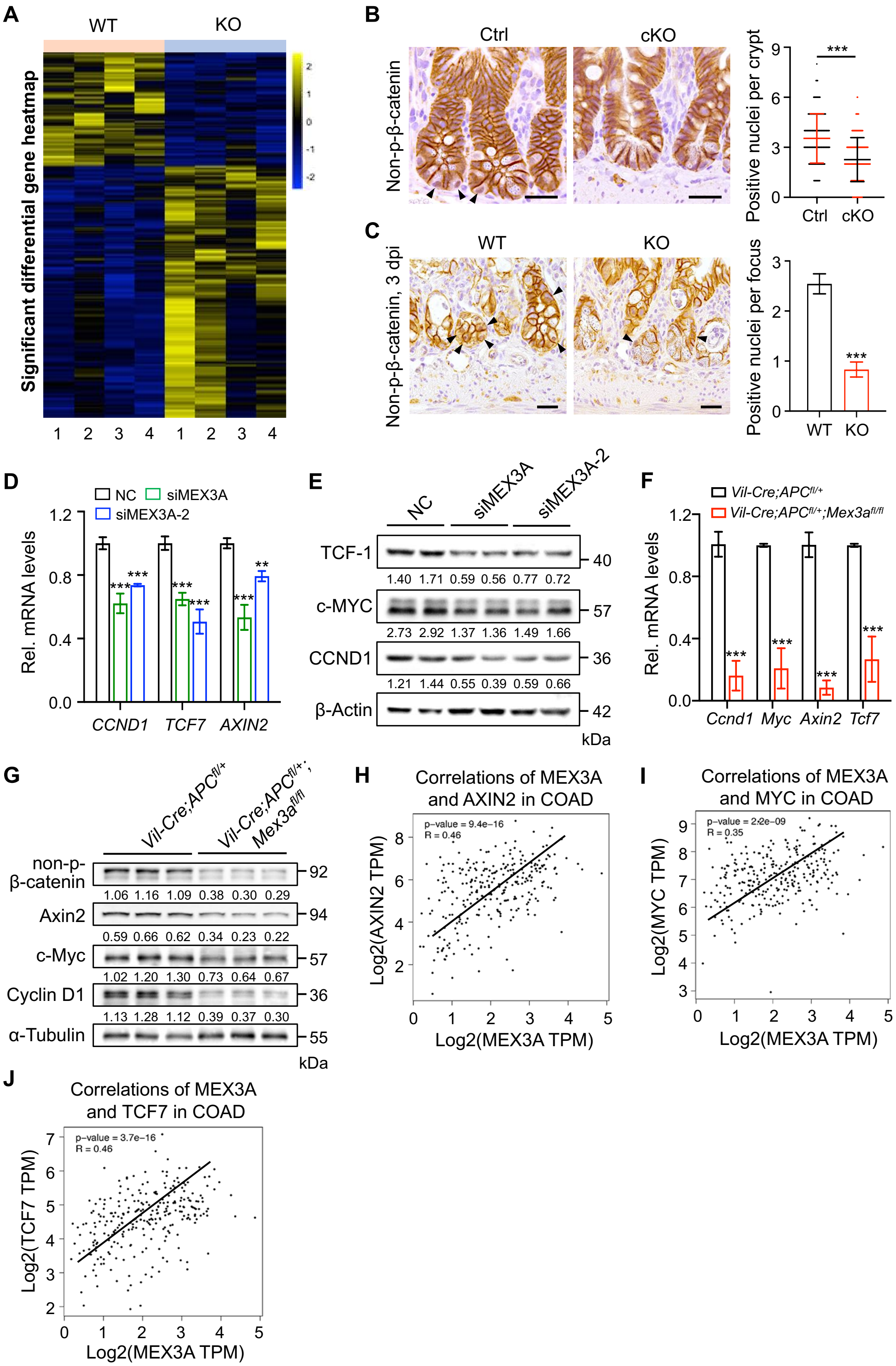
A
WT
KO
Significant differential gene heatmap
1 2 3 4 1 2 3 4
B
Ctrl
cKO
Non-p-β-catenin
Positive nuclei per crypt
***
Ctrl cKO
C
WT
KO
Non-p-β-catenin, 3 dpi
Positive nuclei per focus
***
WT KO
D
NC siMEX3A
siMEX3A-2
Rel. mRNA levels
CCND1 TCF7 AXIN2
E
NC siMEX3A siMEX3A-2
TCF-1
1.40 1.71 0.59 0.56 0.77 0.72
c-MYC
2.73 2.92 1.37 1.36 1.49 1.66
CCND1
1.21 1.44 0.55 0.39 0.59 0.66
β-Actin
kDa
F
Vil-Cre;APCfl/+
Vil-Cre;APCfl/+;Mex3afl/fl
Rel. mRNA levels
Ccnd1 Myc Axin2 Tcf7
G
Vil-Cre;APCfl/+
Vil-Cre;APCfl/+; Mex3afl/fl
non-p-β-catenin
1.06 1.16 1.09 0.38 0.30 0.29
Axin2
0.59 0.66 0.62 0.34 0.23 0.22
c-Myc
1.02 1.20 1.30 0.73 0.64 0.67
Cyclin D1
1.13 1.28 1.12 0.39 0.37 0.30
α-Tubulin
kDa
H
Correlations of MEX3A and AXIN2 in COAD
p-value = 9.4e-16
R = 0.46
Log2(AXIN2 TPM)
Log2(MEX3A TPM)
I
Correlations of MEX3A and MYC in COAD
p-value = 2.2e-09
R = 0.35
Log2(MYC TPM)
Log2(MEX3A TPM)
J
Correlations of MEX3A and TCF7 in COAD
p-value = 3.7e-16
R = 0.46
Log2(TCF7 TPM)
Log2(MEX3A TPM)

Figure S10. Deletion of *Mex3a* suppresses WNT signaling activity. A, Heatmap of differentially expressed genes in transcriptome profiles of intestinal crypts from wild-type (WT) and KO mice. **B,** Immunohistochemistry for non-p-β-catenin in ileum tissues from control (Ctrl) and cKO mice. Black arrowheads point to non-p-β-catenin$^+$ nuclei. Scale bar: 25 µm. Number of nuclear non-p-β-catenin$^+$ cells per crypt was quantified. Ctrl, n = 383 crypts, 3 mice; cKO, n = 398 crypts, 3 mice. **C,** Immunohistochemistry for non-p-β-catenin from WT and KO mice 3 days after 12 Gy γ-radiation. Black arrowheads point to non-p-β-catenin$^+$ nuclei. Number of nuclear non-p-β-catenin$^+$ cells per regenerative focus was quantified. n = 3. Scale bar: 25 µm. **D,** qRT-PCR analysis of WNT target genes *CCND1*, *TCF7* and *AXIN2* in HCT116 cells transfected with *MEX3A* siRNAs (siMEX3As). n = 3. **E,** Western blotting for CCND1, c-MYC and TCF-1 in HCT116 cells transfected with siMEX3As and negative control. β-Actin was used as a loading control. **F,** qRT-PCR analysis of WNT target genes in intestinal tumors from *Vil-Cre;APC$^{fl/+}$* and *Vil-Cre;APC$^{fl/+}$;Mex3a$^{fl/fl}$* mice at 4 months of age. n = 3. **G,** Western blotting for Cyclin D1, c-Myc, Axin2 and non-p-β-catenin in intestinal tumors from *Vil-Cre;APC$^{fl/+}$* and *Vil-Cre;APC$^{fl/+}$;Mex3a$^{fl/fl}$* mice at 4 months of age. α-Tubulin was used as a loading control. **H-J,** Spearman correlation analysis of MEX3A and AXIN2 ($P < 0.001$; $R = 0.46$) in panel H, MEX3A and MYC ($P < 0.001$; $R = 0.35$) in panel I, MEX3A and TCF7 ($P < 0.001$; $R = 0.46$) in panel J, in human CRC based on TCGA database. Data are presented as the mean ± SD. *$P < 0.05$; **$P < 0.01$; ***$P < 0.001$.

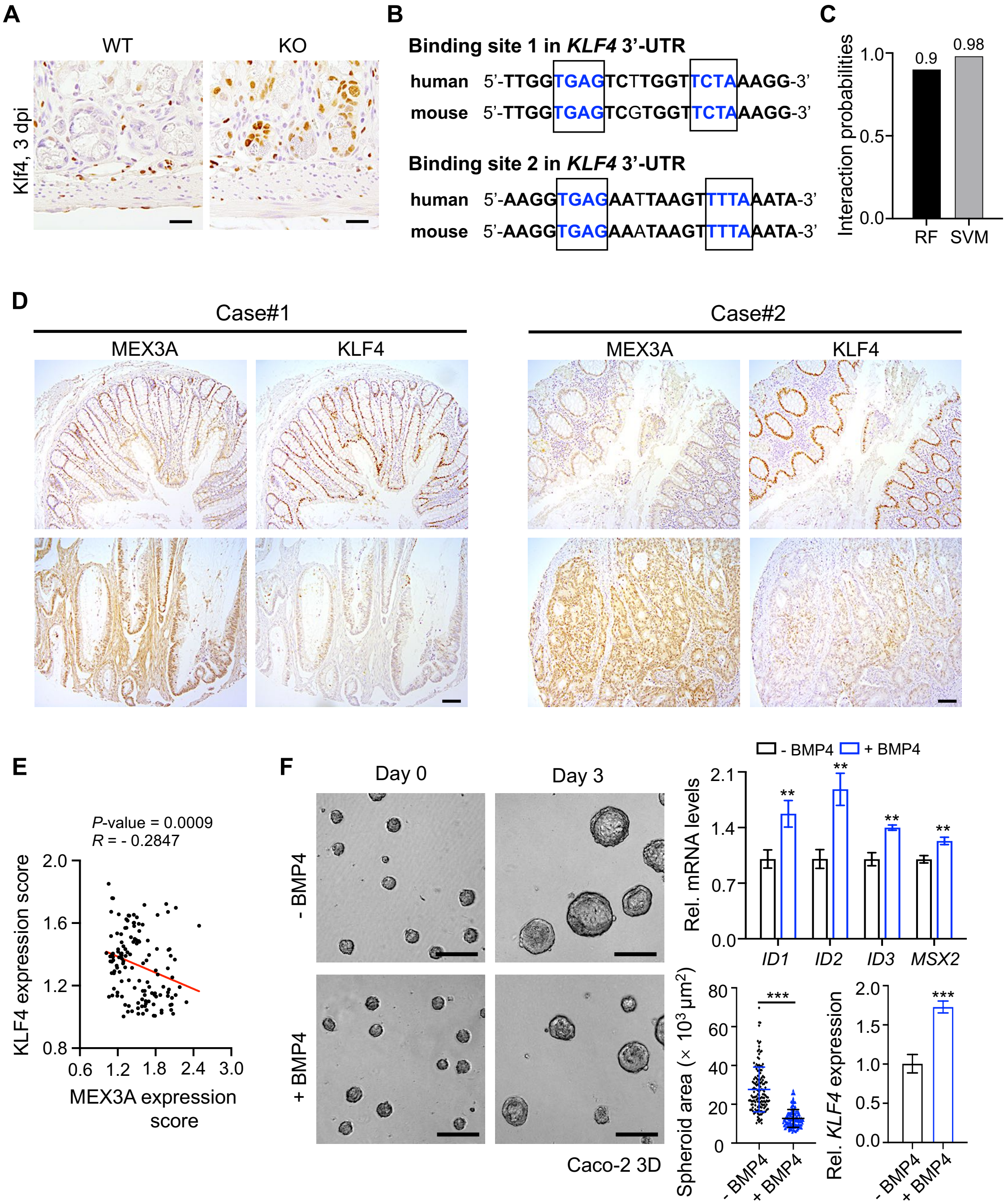

Figure S11. *KLF4* acts as a direct target of MEX3A in the intestine. A, Representative immunohistochemical images of Klf4 in intestinal regenerative foci from wild-type (WT) and KO mice 3 days postirradiation. Scale bar: 25 μm. **B,** MEX3A binding sites are located in the *KLF4* 3'-UTR region that are conserved between human and mouse. **C,** Interaction probability between MEX3A and *KLF4* was predicted by RPISeq. RF = 0.9, SVM = 0.98. **D,** Immunohistochemical staining for MEX3A and KLF4 in a tissue array containing 66 paired CRC tumor and peri-tumor tissues. Scale bar: 100 μm. **E,** Spearman correlation analysis of MEX3A and KLF4 expression scores (P = 0.0009; R = -0.2847) in CRC tissue array in panel D. **F,** Representative images of

spheroids for 3D cultured Caco-2 cells upon BMP4 treatment. Scale bar: 200 µm. Spheroids were grown for 72 hours and then treated with 50 ng/mL BMP4 for 72 h. BMP target genes (*ID1*, *ID2*, *ID3* and *MSX2*) and *KLF4* expression in 3D cultured Caco-2 cells treated with BMP4 were measured by qRT-PCR. Spheroid area was quantified. n = 3 technical replicates. Data are presented as the mean ± SD. *$P < 0.05$; **$P < 0.01$; ***$P < 0.001$.

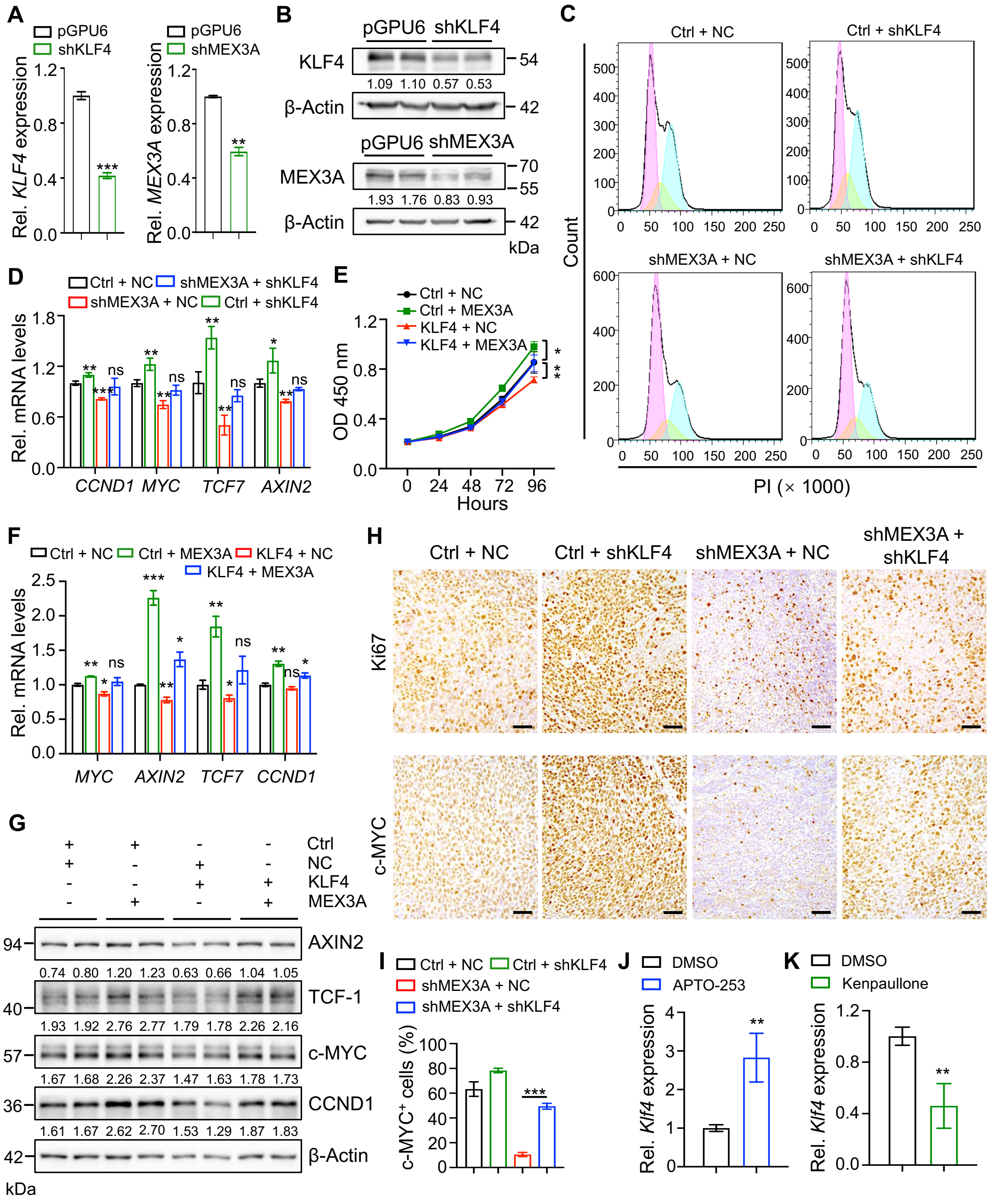

Figure S12. MEX3A-mediated hyperproliferation phenotypes can be rescued by suppression of *KLF4*. A, qRT-PCR analysis of *KLF4* and *MEX3A* in HCT116 cells transfected with shKLF4 or shMEX3A plasmids. n = 3. **B,** Western blotting of KLF4 and MEX3A in HCT116 cells transfected with shKLF4 or shMEX3A plasmids. β-Actin was used as a loading control. **C,** Cell cycle distribution of HCT116 cells transfected with shMEX3A and/or shKLF4. n = 3. **D,** qRT-PCR analysis of WNT target genes *CCND1*, *MYC*, *TCF7* and *AXIN2* in HCT116 cells transfected with shMEX3A and/or shKLF4. n = 3. **E,** Growth curve of HCT116 cells transfected with MEX3A

and/or KLF4 plasmids over time. n = 4. **F**-**G,** qRT-PCR (**F**) and Western blotting (**G**) analysis of WNT target genes *CCND1*/CCND1, *MYC*/c-MYC, *TCF7*/TCF-1 and *AXIN2*/AXIN2. β-Actin was used as loading control. **H,** Immunohistochemical staining for Ki67 and c-MYC in xenografted tumors from HCT116 cells transfected with shMEX3A and/or shKLF4. n = 6. Scale bar: 50 µm. **I,** Quantification of c-MYC^{+} cell percentages in panel H. **J**-**K,** qRT-PCR for *Klf4* in mouse tumor organoids treated with APTO-253 (**J**) or kenpaullone (**K**) for 72 hours following 48 hours of culture. n = 3. Data are presented as the mean ± SD. *$P < 0.05$; **$P < 0.01$; ***$P < 0.001$.

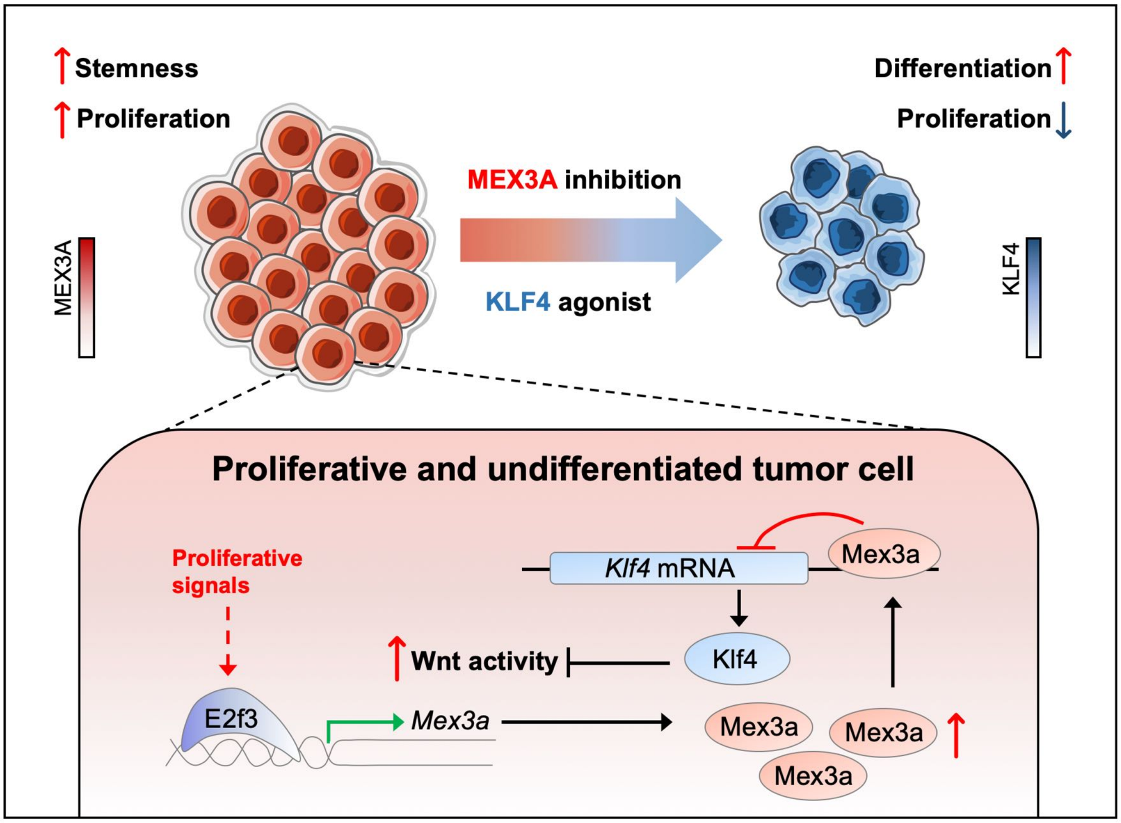

Figure S13. The working model of E2F3-MEX3A-KLF4 axis in driving intestinal tumorigenicity.

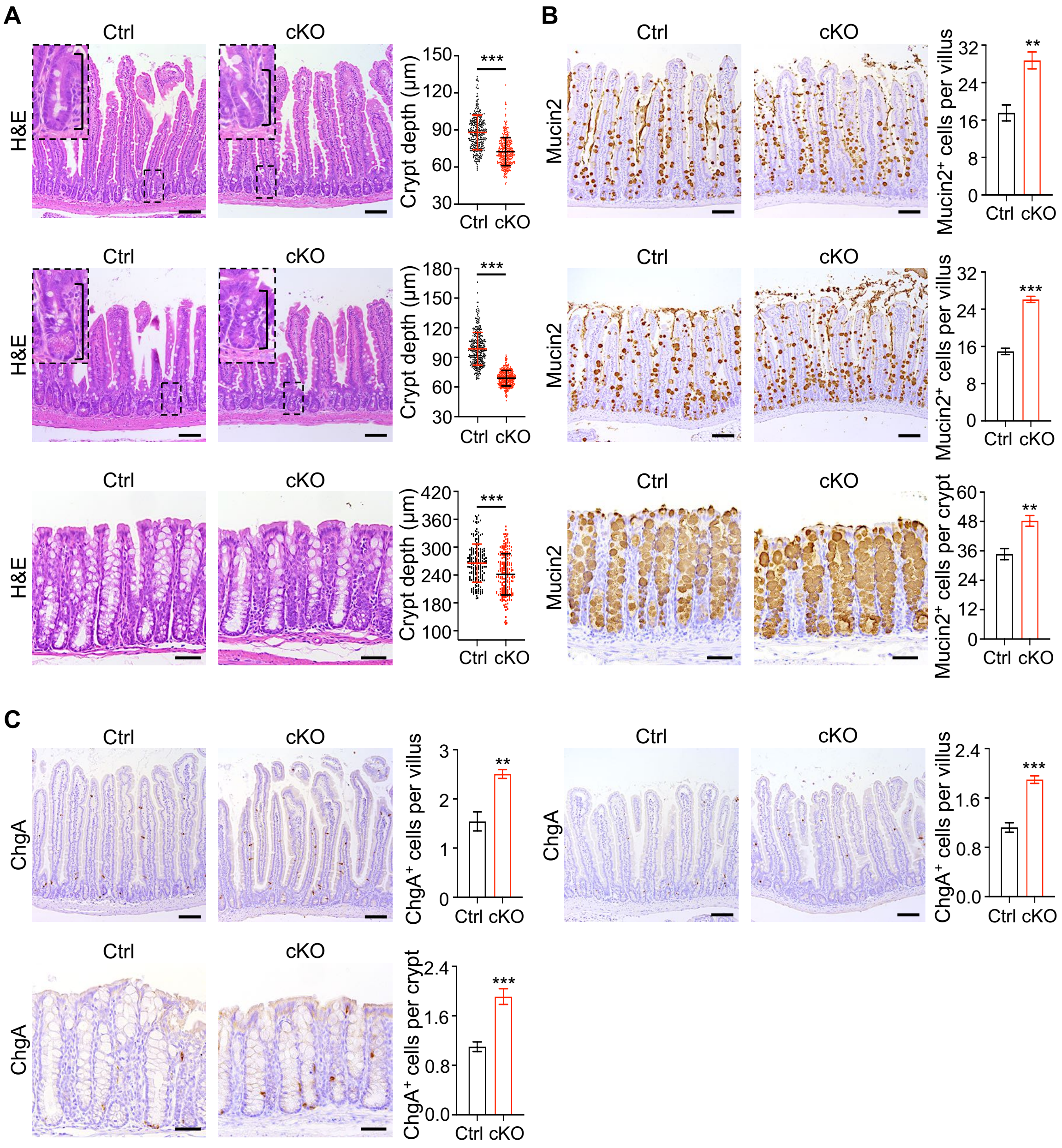

Figure EV1. Deletion of *Mex3a* in different regions of intestine results in disrupted intestinal homeostasis. A, Histology of duodenum, jejunum and colon from *Villin-Cre;Mex3a*$^{fl/fl}$ (cKO) and littermate control (Ctrl) mice. Crypt depth was quantified. Ctrl, n = 326 crypts in duodenum, n = 361 crypts in jejunum, n = 198 crypts in colon, n = 3 mice; cKO, n = 365 crypts in duodenum, n = 368 crypts in jejunum, n = 193 crypts in colon, n = 3 mice. Scale bar: 100 μm for duodenum and jejunum, 50 μm for colon. **B-C,** Immunohistochemistry for Mucin2 (**B**) and ChgA (**C**) in duodenum, jejunum and colon from Ctrl and cKO mice. Mucin2^{+} cells and ChgA^{+} cells per crypt-villus architecture were quantified. n = 3. Scale bar: 100 μm for duodenum and jejunum, 50 μm for colon.

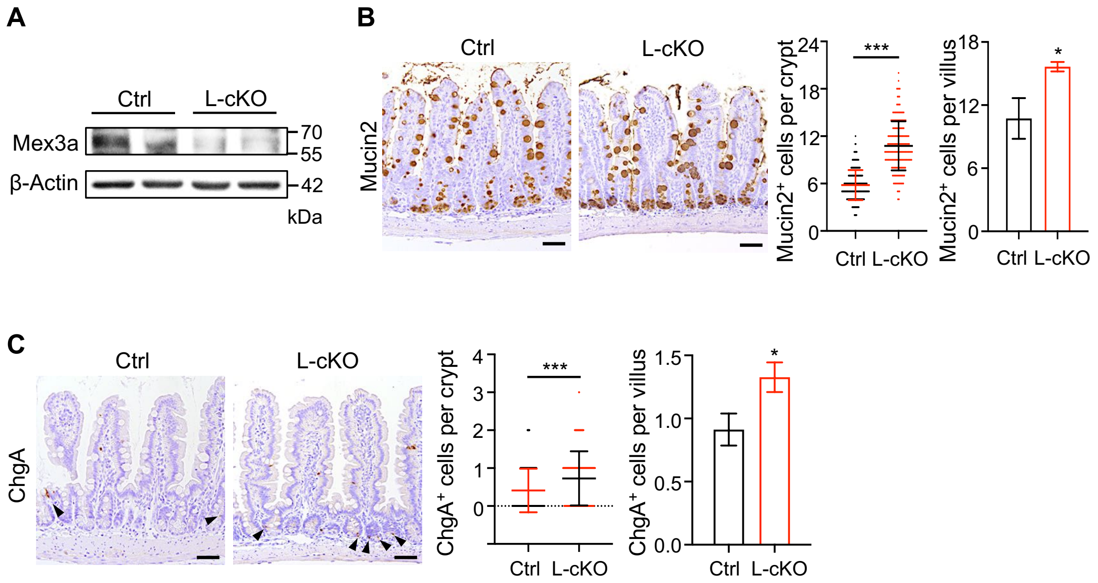

Figure EV2. Deletion of *Mex3a* in Lgr5$^+$ ISCs exacerbates differentiation. A, Western blotting for Mex3a in intestinal tissues from $Lgr5^{EGFP\text{-}CreERT2}$;$Mex3a^{fl/fl}$ (L-cKO) and littermate control (Ctrl) mice. β-Actin was used as a loading control. **B,** Immunohistochemistry for Mucin2 in ileum tissues from Ctrl and L-cKO mice. Number of Mucin2$^+$ cells per crypt and per villus were quantified. Ctrl, n = 221 crypts, 3 mice; L-cKO, n = 199 crypts, 3 mice. Scale bar: 50 μm. **C,** Immunohistochemistry for ChgA and quantification of ChgA$^+$ cells per crypt and per villus in ileum from Ctrl and L-cKO mice. Ctrl, n = 229 crypts, 3 mice; L-cKO, n = 214 crypts, 3 mice. Scale bar: 50 μm.

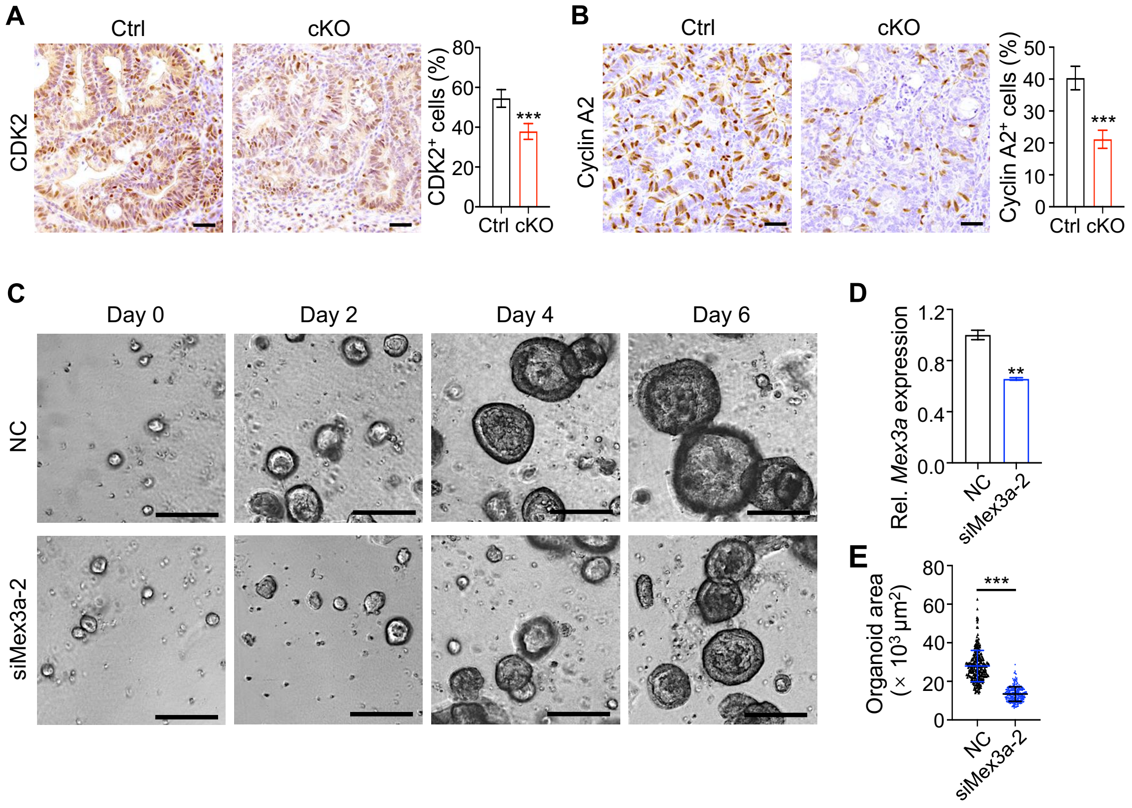

Figure EV3. Depletion of *Mex3a* suppresses proliferation of tumor cells. A-B, Immunohistochemistry for CDK2 (**A**) and Cyclin A2 (**B**) in AOM-DSS colon tumors from control (Ctrl) and *Mex3a* cKO mice. Percentage of $CDK2^{+}$ cells and Cyclin $A2^{+}$ cells were quantified. n = 7. Scale bar: 50 µm. **C,** Growth of APKS mouse tumor organoids over time. The organoids were transfected with siMex3a-2. n = 3. Scale bar: 200 µm. **D,** qRT-PCR analysis of *Mex3a* in mouse tumor organoids after transfection with siMex3a-2. n = 3. **E,** Quantification of the organoid area in panel C.

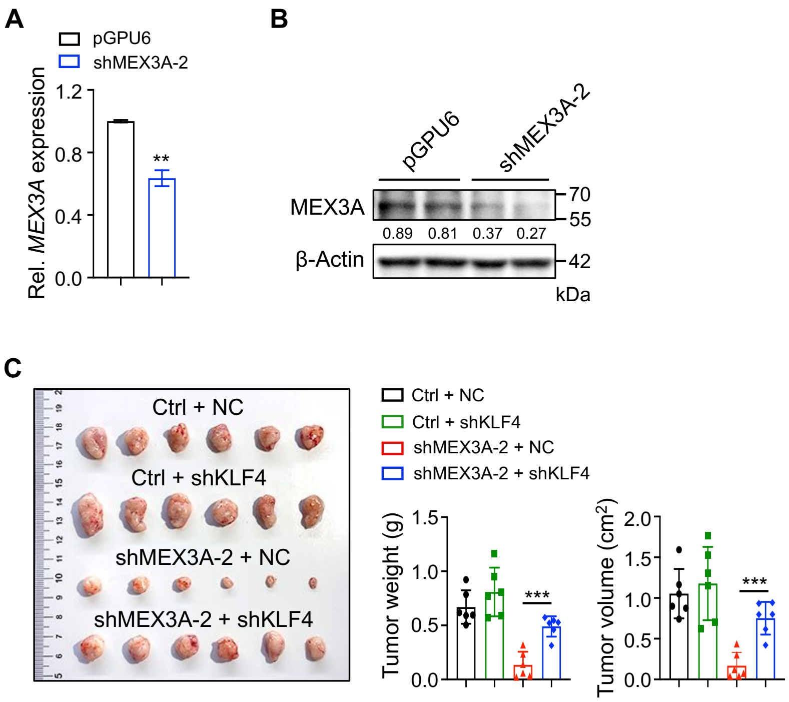

Figure EV4. *KLF4* knockdown rescues *MEX3A* inhibition-induced suppression of tumor growth. A, qRT-PCR analysis of *MEX3A* in HCT116 cells transfected with shMEX3A-2 plasmid. n = 3. **B,** Western blotting of MEX3A in HCT116 cells transfected with shMEX3A-2 plasmids. β-Actin was used as a loading control. **C,** Gross images of xenografted tumors 3 weeks after transplantation with shMEX3A-2 and/or shKLF4-transfected HCT116 cells. Tumor weight and volume were quantified. n = 6.

Table S1. Colorectal cancer tissue microarray information (NO. T17-952 TMA4)

Position	Patient ID	Gender	Age	Location	AJCC stage	T stage	N stage	M stage	Histology	Grade	Venous invasion	Perineural invasion
A03/A04	290891	male	74	Ascending Colon	II	4a	0	0	Adenocarcinoma	G2	(-)	(-)
A05/A06	289871	female	56	Rectum	II	4a	0	0	Adenocarcinoma	G2	(+)	(-)
A07/A08	289926	male	65	Sigmoid Colon	II	4a	0	0	Adenocarcinoma	G2	(-)	(-)
A09/A10	291976	male	54	Rectum	II	3	0	0	Adenocarcinoma	G2	(-)	(+)
A11/A12	289521	male	75	Rectum	II	3	0	0	Adenocarcinoma	G2	(+)	(-)
A15/A16	288072	female	43	Rectum	II	3	0	0	Adenocarcinoma	G2	(+)	(-)
B01/B02	288639	female	70	Sigmoid Colon	II	3	0	0	Mucinous Adenocarcinoma	G3	(-)	(-)
B03/B04	287882	female	57	Ascending Colon	II	4a	0	0	Adenocarcinoma	G2	(-)	(+)
B05/B06	131752	male	60	Rectum	II	3	0	0	Adenocarcinoma	G2	(-)	(+)
B07/B08	287303	female	45	Descending Colon	II	3	0	0	Adenocarcinoma	G1	(-)	(-)
B09/B10	286725	male	46	Rectum	II	3	0	0	Adenocarcinoma	G2	(-)	(+)
B11/B12	286535	male	51	Sigmoid Colon	II	4a	0	0	Adenocarcinoma	G2	(-)	(-)
B15/B16	285765	male	58	Rectum	II	3	0	0	Adenocarcinoma	G2	(-)	(+)
C01/C02	285472	male	80	Ascending Colon	II	4a	0	0	Adenocarcinoma	G2	(-)	(-)
C03/C04	285579	male	70	Rectum	II	3	0	0	Adenocarcinoma	G2	(-)	(-)
C05/C06	281750	female	58	Ascending Colon	II	3	0	0	Adenocarcinoma	G2	(-)	(-)

C07/C08	284256	female	48	Transverse Colon	II	3	0	0	Mucinous Adenocarcinoma	G2	(-)	(-)
C09/C10	284522	female	68	Ascending Colon	II	3	0	0	Mucinous Adenocarcinoma	G3	(-)	(-)
C11/C12	282429	female	55	Ascending Colon	II	3	0	0	Mucinous Adenocarcinoma	G2	—	(-)
C13/C14	284164	male	47	Rectum	II	3	0	0	Adenocarcinoma	G2	(-)	(-)
C15/C16	282439	female	65	Rectum	II	3	0	0	Adenocarcinoma	G2	—	(+)
D01/D02	282474	male	62	Rectum	II	4	0	0	Adenocarcinoma	G2	(-)	(-)
D03/D04	282439	female	65	Rectum	II	3	0	0	Adenocarcinoma	G2	—	(+)
D05/D06	281977	male	55	Ascending Colon	II	4a	0	0	Adenocarcinoma	G2	(-)	(-)
D07/D08	279559	male	61	Rectum	II	3	0	0	Adenocarcinoma	G2	(-)	(-)
D09/D10	277582	male	56	Transverse Colon	II	4a	0	0	Adenocarcinoma	G2	(-)	(-)
D11/D12	277736	female	68	Descending Colon	II	3	0	0	Adenocarcinoma	G2	(-)	(-)
D13/D14	277476	female	64	Rectum	II	3	0	0	Mucinous Adenocarcinoma	—	(-)	(-)
D15/D16	277477	male	74	Sigmoid Colon	II	4a	0	0	Adenocarcinoma	G2	(-)	(-)
E01/E02	277990	male	46	Sigmoid Colon	II	4a	0	0	Adenocarcinoma	G2	(-)	(-)
E03/E04	277974	male	33	Descending Colon	II	3	0	0	Adenocarcinoma	G2	(-)	(-)
E05/E06	278727	female	56	Rectum	II	3	0	0	Adenocarcinoma	G2	(-)	(+)
E07/E08	278189	male	55	Descending Colon	II	4a	0	0	Adenocarcinoma	G2	(-)	(-)
E09/E10	277418	male	54	Rectum	II	3	0	0	Adenocarcinoma	G2	(-)	(-)
E11/E12	277357	female	69	Sigmoid Colon	II	4	0	0	Adenocarcinoma	G2	(-)	(-)

E13/E14	276367	female	53	Rectum	II	3	0	0	Adenocarcinoma	G2	(-)	(-)
E15/E16	276473	female	58	Sigmoid Colon	II	4	0	0	Adenocarcinoma	G2	(-)	(+)
F01/F02	276398	male	63	Rectum	II	3	0	0	Adenocarcinoma	G2	(+)	(-)
F03/F04	275877	male	67	Descending Colon	II	4a	0	0	Adenocarcinoma	G2	(-)	(-)
F05/F06	275261	male	56	Ascending Colon	II	3	0	0	Adenocarcinoma	G2	(-)	(+)
F07/F08	275397	female	71	Rectum	II	4b	0	0	Adenocarcinoma	G2	(-)	(-)
F09/F10	274836	female	77	Rectum	II	3	0	0	Adenocarcinoma	G2	(-)	(+)
F11/F12	275536	male	58	Ascending Colon	II	4a	0	0	Adenocarcinoma	G2	(-)	(-)
F13/F14	274433	male	61	Rectum	II	3	0	0	Adenocarcinoma	G2	(-)	(-)
F15/F16	273985	male	39	Rectum	II	3	0	0	Adenocarcinoma	G2	(-)	(-)
G01/G02	273560	female	50	Rectum	II	3	0	0	Adenocarcinoma	G2	(-)	(+)
G03/G04	273299	female	36	Rectum	II	3	0	0	Adenocarcinoma	G2	(-)	(+)
G05/G06	266296	male	69	Rectum	II	3	0	0	Adenocarcinoma	G2	(-)	(-)
G07/G08	273037	female	63	Descending Colon	II	3	0	0	Adenocarcinoma	G2	(+)	(-)
G09/G10	271983	male	72	Rectum	II	3	0	0	Adenocarcinoma	G2	(-)	(-)
G11/G12	163173	female	65	Sigmoid Colon	II	4a	0	0	Adenocarcinoma	G2	(-)	(-)
G13/G14	271902	female	39	Ascending Colon	II	4	0	0	Adenocarcinoma	G2	(+)	(-)
G15/G16	249097	female	67	Sigmoid Colon	II	4a	0	0	Adenocarcinoma	G1	(-)	(-)
H01/H02	248689	male	55	Rectum	II	3	0	0	Mucinous Adenocarcinoma	—	(+)	(+)
H03/H04	249543	male	65	Ascending Colon	II	3	0	0	Adenocarcinoma	G2	(-)	(-)

H05/H06	255405	male	65	Sigmoid Colon	II	3	0	0	Mucinous Adenocarcinoma	—	(-)	(-)
H07/H08	255371	male	55	Ascending Colon	II	3	0	0	Adenocarcinoma	G2	—	(-)
H09/H10	255410	female	38	Rectum	II	3	0	0	Adenocarcinoma	G2	(-)	(-)
H11/H12	257685	male	37	Ascending Colon	II	4a	0	0	Adenocarcinoma	G2	(-)	(-)
H13/H14	257957	female	78	Rectum	II	3	0	0	Adenocarcinoma	G2	(-)	(+)
H15/H16	261421	male	71	Ascending Colon	II	4a	0	0	Mucinous Adenocarcinoma	—	(-)	(-)
I01/I02	259507	female	79	Ascending Colon	II	4a	0	0	Adenocarcinoma	G2	(-)	(-)
I03/I04	262266	male	55	Ascending Colon	II	3	0	0	Adenocarcinoma	G1	(-)	(-)
I05/I06	261726	female	55	Sigmoid Colon	II	3	0	0	Adenocarcinoma	G2	(-)	—
I07/I08	261840	female	53	Descending Colon	II	3	0	0	Mucinous Adenocarcinoma	—	(-)	(-)
I09/I10	261811	female	53	Rectum	II	3	0	0	Adenocarcinoma	G2	(+)	(-)
I11/I12	264959	female	51	Rectum	II	3	0	0	Adenocarcinoma	G2	(-)	(-)
I13/I14	264843	male	42	Transverse Colon	II	4	0	0	Adenocarcinoma	G2	(-)	(-)
I15/I16	265053	female	61	Sigmoid Colon	II	4a	0	0	Adenocarcinoma	G2	(+)	(+)
J01/J02	265941	female	65	Rectum	II	3	0	0	Adenocarcinoma	G2	(-)	(-)
J03/J04	266117	male	55	Rectum	II	3	0	0	Adenocarcinoma	G2	(-)	(-)
J05/J06	266225	female	43	Transverse Colon	II	4	0	0	Adenocarcinoma	G2	(-)	(-)
J07/J08	266000	male	57	Sigmoid Colon	II	3	0	0	Adenocarcinoma	G1	(-)	(-)
J09/J10	266752	male	58	Sigmoid Colon	II	4a	0	0	Adenocarcinoma	G2	(-)	(+)

J11/J12	267082	male	76	Sigmoid Colon	II	4a	0	0	Adenocarcinoma	G2	(-)	(+)
J13	268385	female	64	Sigmoid Colon	II	3	0	0	Adenocarcinoma	G2	(-)	(-)
J14	246235	male	50	Rectum	II	3	0	0	Adenocarcinoma	G2	(-)	(-)
J15	244442	male	59	Rectum	II	3	0	0	Mucinous Adenocarcinoma	G1	(-)	(-)
J16	385156	male	79	Rectum	II	3	0	0	Adenocarcinoma	G2	(-)	(+)
K01	449311	female	66	Sigmoid Colon	II	3	0	0	Adenocarcinoma	G2	(-)	(-)
K02	289816	male	66	Rectum	II	3	0	0	Adenocarcinoma	G1	(-)	(+)
K04	286595	female	52	Rectum	II	3	0	0	Adenocarcinoma	G2	(-)	(-)
K05	282416	male	37	Sigmoid Colon	II	4a	0	0	Mucinous Adenocarcinoma	G3	(-)	(-)
K07	258957	female	57	Rectum	II	3	0	0	Adenocarcinoma	G2	(-)	(-)
K08	261702	male	24	Ascending Colon	II	3	0	0	Adenocarcinoma	G1	(-)	(-)
K09	266446	male	58	Descending Colon	II	4a	0	0	Adenocarcinoma	G2	(-)	(-)

Table S2. qRT-PCR primers

Genes	Forward Primer 5'-3'	Reverse Primer 5'-3'	Application
Gadph	GTGCCGCCTGGAGAAACCT	AAGTCGCAGGAGACAACC	qRT-PCR
Lgr5	CAGCCTCAAAGTGCTTATGCT	GTGGCACGTAACTGATGTGG	qRT-PCR
Mex3a	ACACCACGGAGTGCGTTC	GTTGGTTTTGGCCCTCAGA	qRT-PCR
E2f3	AAACGCGGTATGATACGTCCC	CCATCAGGAGACTGGCTCAG	qRT-PCR
Tnfrsf19	TTCTGTGGGGGACACGATG	AGAAAATTCAGCGCAGATGGAA	qRT-PCR
Ascl2	AAGCACACCTTGACTGGTACG	AAGTGGACGTTTGCACCTTCA	qRT-PCR
Smoc2	AGTGGAGACATTGGCAAGAAG	ACACACTTTTTGGGCTTGGATT	qRT-PCR
Mki67	GCTGTCCTCAAGACAATCATCA	GGCGTTATCCCAGGAGACT	qRT-PCR
Axin2	TGCCGACCTCAAGTGCA	ACGCTACTGTCCGTCATGG	qRT-PCR
Ccnd1	ATTGTGCCATCCATGCG	TAGATGCACAACTTCTCGGC	qRT-PCR
Myc	TAGTGCTGCATGAGGAGACA	CATCAATTTCTTCCTCATCTTC	qRT-PCR
Frat2	GTGGCTTCTCACCGAATCCAG	AGTGACTGAGTCCGGTCCG	qRT-PCR
Fzd2	GCCGTCCTATCTCAGCTATAAGT	TCTCCTCTTGCGAGAAGAACATA	qRT-PCR
Znrf3	GGCGACTATACCACCCACAC	CTTCACCACTCCTACCCAGC	qRT-PCR
Tcf7	CTGCCTGCTCACAGTTCC	GGCTCCAGGCCTGTGG	qRT-PCR
Klf4	GCACACCTGCGAACTCACAC	CCGTCCCAGTCACAGTGGTAA	qRT-PCR
GAPDH	GGAGCGAGATCCCTCCAAAAT	GGCTGTTGTCATACTTCTCATGG	qRT-PCR
MEX3A	CAAGCTCTGCGCTCTCTACAAA	GGCCTTAATCTTGCAGCCTTG	qRT-PCR
E2F3	AAAGCCCCTCCAGAAACAAGA	CCTTGGGTACTTGCCAAATGT	qRT-PCR
CCND1	TGCCACAGATGTGAAGTTCATT	CAGTCCGGGTCACACTTGAT	qRT-PCR
TCF7	TGGCTTCTACTCCCTGACCT	TCTCTGCCTTCCACTCTGCT	qRT-PCR
AXIN2	CAAGGGCCAGGTCACCAA	CCCCCAACCCATCTTCGT	qRT-PCR
MYC	CACCAGCAGCGACTCTGA	GATCCAGACTCTGACCTTTTGC	qRT-PCR
KLF4	AGGGAGAAGACACTGCGTCA	ACGATCGTCTTCCCCTCTTT	qRT-PCR
ID1	GCTGTTACTCACGCCTCAAG	CAACTGAAGGTCCCTGATGTAG	qRT-PCR
ID2	ACTGCTACTCCAAGCTCAAGG	TGCAGGTCCAAGATGTAGTCG	qRT-PCR
ID3	AATCCTACAGCGCGTCATC	TGTCTGGATGGGAAGGTG	qRT-PCR
MSX2	CGGTCAAGTCGGAAAATTCAG	CGAATATCGGCCGGGTTC	qRT-PCR
CDX2	TGTTGTTGTTGCTGCTGT	AATACTCCCCACTTCCCT	CLIP-qPCR
KLF4	TAGCCTAAATGATGGTGC	CATAAATGTTGATCGGAAG	CLIP-qPCR
E2f3-1	AACCCACCCGAGGCTTTT	TGCCGGGAGTTGTAGTTTCC	ChIP-qPCR
E2f3-2	CGAGCCCGTGGACTC	GCGTTTCTCCTCTGCC	ChIP-qPCR

Table S3. Primers used in subcloning of short-hairpin RNA into the pGPU6-GFP vector

shRNA	Direction	Sequence
sh*MEX3A*	Forward	CACCGCGGACTCTGGCTTTGTTCAAGAGACAAAGCCAGAGTCCACTCCGCTTTTTTG
	Reverse	GATCCAAAAAAGCGGAGTGGACTCTGGCTTTGTCTCTTGAACAAAGCCAGAGTCCACTCCGC
sh*MEX3A*-2	Forward	CACCGCCACATCACAGCCACGCAAGCTTCAAGAGAGCTTGCGTGGCTGTGATGTGGTTTTTTG
	Reverse	GATCCAAAAAACCACATCACAGCCACGCAAGCTCTCTTGAAGCTTGCGTGGCTGTGATGTGGC
sh*KLF4*	Forward	CACCGGACGGCTGTGGATGGAAATTTCAAGAGAATTTCCATCCACAGCCGTCCTTTTTTG
	Reverse	GATCCAAAAAAGGACGGCTGTGGATGGAAATTCTCTTGAAATTTCCATCCACAGCCGTCC

Table S4. siRNA sequence of MEX3A

Genes	Sequence (5'-3')
siMEX3A	GCGGAGUGGACUCUGGCUU
siMEX3A-2	GCUACGGCGGGUACCUCUU
siMex3a	GCAGCAGACCAACACGUAC
siMex3a-2	GCCACACAAGCCAUCCGAA
